# Plasmidome of *Listeria* spp.—The *repA*-Family Business

**DOI:** 10.3390/ijms221910320

**Published:** 2021-09-25

**Authors:** Cora Chmielowska, Dorota Korsak, Elvira Chapkauskaitse, Przemysław Decewicz, Robert Lasek, Magdalena Szuplewska, Dariusz Bartosik

**Affiliations:** 1 Department of Bacterial Genetics, Institute of Microbiology, Faculty of Biology, University of Warsaw, Miecznikowa 1, 02-096 Warsaw, Poland; e.chapkauskait@student.uw.edu.pl (E.C.); lasek@biol.uw.edu.pl (R.L.); mszuplewska@biol.uw.edu.pl (M.S.); 2 Department of Molecular Microbiology, Institute of Microbiology, Faculty of Biology, University of Warsaw, Miecznikowa 1, 02-096 Warsaw, Poland; d.korsak@uw.edu.pl; 3 Department of Environmental Microbiology and Biotechnology, Institute of Microbiology, Faculty of Biology, University of Warsaw, Miecznikowa 1, 02-096 Warsaw, Poland; decewicz@biol.uw.edu.pl

**Keywords:** *Listeria*, plasmids, adaptation, heavy metal resistance, plasmidome, pangenome, *Firmicutes*, horizontal gene transfer

## Abstract

Bacteria of the genus *Listeria* (phylum *Firmicutes*) include both human and animal pathogens, as well as saprophytic strains. A common component of *Listeria* spp. genomes are plasmids, i.e., extrachromosomal replicons that contribute to gene flux in bacteria. This study provides an in-depth insight into the structure, diversity and evolution of plasmids occurring in *Listeria* strains inhabiting various environments under different anthropogenic pressures. Apart from the components of the conserved plasmid backbone (providing replication, stable maintenance and conjugational transfer functions), these replicons contain numerous adaptive genes possibly involved in: (i) resistance to antibiotics, heavy metals, metalloids and sanitizers, and (ii) responses to heat, oxidative, acid and high salinity stressors. Their genomes are also enriched by numerous transposable elements, which have influenced the plasmid architecture. The plasmidome of *Listeria* is dominated by a group of related replicons encoding the RepA replication initiation protein. Detailed comparative analyses provide valuable data on the level of conservation of these replicons and their role in shaping the structure of the *Listeria* pangenome, as well as their relationship to plasmids of other genera of *Firmicutes*, which demonstrates the range and direction of flow of genetic information in this important group of bacteria.

## 1. Introduction

The genus *Listeria* (*Firmicutes*) comprises 26 species, but studies have mainly focused on only one, *L. monocytogenes*, a pathogenic bacterium causing rare but severe food poisoning that can be fatal to unborn children and individuals with a compromised immune system [1]. *Listeria* spp. strains are ubiquitous in natural and agricultural environments. They are also commonly found in different food processing scenarios where they can tolerate a wide range of stress conditions, such as the presence of sanitizers, high salinity, acidity and non-optimal temperatures (e.g., [2,3,4,5]).

Many genes that may contribute to the ability of *Listeria* spp. to colonize various environments have been identified within mobile genetic elements, mainly plasmids. These genes can confer resistance to antibiotics [6,7], sanitizers [8,9], heavy metals [10,11,12] or elevated temperatures [13]. Several studies have identified plasmids that are commonly present and highly conserved among *Listeria* strains isolated from various sources and different geographic locations, which suggests a strong selection pressure for the maintenance of these replicons [12,14,15,16,17,18,19].

Recently, a large-scale survey of plasmid sequences extracted from whole genome sequencing (WGS) data from almost 2000 *L. monocytogenes* strains was performed by Schmitz-Esser and colleagues [15]. This study indicated that plasmids are common in this species (54% of tested strains harbored such replicons), although their distribution varied greatly depending on the *L. monocytogenes* sequence type (ST) and lineage. WGS data are a valuable source of information on the distribution and diversity of bacterial plasmids, but they provide limited information on the structure, genetic load and evolution of these replicons due to the often incomplete sequences obtained.

Little is known about plasmids occurring in *Listeria* species other than *L. monocytogenes*, even though the conjugative transfer of replicons conferring heavy-metal and sanitizer resistance between nonpathogenic and pathogenic species of this genus has been documented [20,21,22]. Apart from the plasmid sequences obtained in our laboratory, 56 unique complete plasmid nucleotide sequences from *L. monocytogenes* were present in GenBank (NCBI) as of 6 December 2020, compared to only three from *L. innocua* and one from *L. grayi*.

In the present study, we obtained the complete nucleotide sequences of 50 plasmids isolated from strains representing six *Listeria* species, originating from natural and food processing environments. Combined with data available in GenBank and from our previous studies, we analyzed a set of 113 complete plasmid sequences from different *Listeria* species to provide an in-depth insight into their diversity, genetic load and potential contribution to the stress responses of these bacteria, as well as the impact of transposable elements (TEs) on the evolution of these replicons.

## 2. Results and Discussion

### 2.1. Identification and Classification of Plasmids Occurring in 782 Listeria spp. Strains

We gathered a large collection of *Listeria* spp. strains, isolated from various environments, that are subject to different anthropogenic pressures. In our previous studies [4,20,23,24] we analyzed the plasmid content of 566 strains. In the present study, the plasmid profiles of an additional 216 strains were determined. The entire collection of 782 strains represents seven *Listeria* species: *L. monocytogenes* (526 strains), *L. innocua* (105), *L. seeligeri* (71), *L. welshimeri* (59), *L. ivanovii* (16), *L. marthii* (4) and *L. grayi* (1) (Supplementary Table S1). The majority of the strains (627) originated from various food and food production-associated sources, while the others (155) came from environmental soil and water samples. The strains carried 374 plasmids in total: 18 small replicons (<10 kb; present in 2% of tested strains) and 356 larger replicons (>15 kb; harbored by 46% of strains). Only four strains maintained two plasmids each.

**Figure 1:**
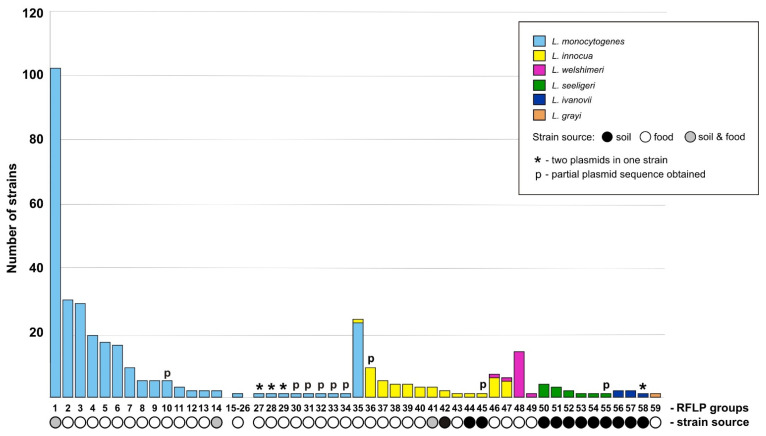
Classification of *Listeria* spp. plasmids identified in our studies according to their restriction fragment length polymorphism (RFLP) profiles, arranged by species and in descending order of the number of strains with identical plasmid profiles.

The *Listeria* spp. strains were classified into 59 groups based on their plasmid restriction fragment length polymorphism (RFLP) patterns (Figure 1, Table S1). The highest numbers of plasmid RFLP profiles were found in strains of *L. monocytogenes* (35 groups) and *L. innocua* (13 groups), i.e., the two most numerous species in our strain collection. In three cases, plasmids with the same RFLP profile were observed in strains of different species, suggesting their horizontal transmission (Figure 1). The majority of RFLP profiles were found only in single strains. However, the most common plasmid group was present in as many as 102 *L. monocytogenes* strains. Plasmids with identical RFLP profiles were found in strains originating from both food-associated and environmental sources in only three cases (Figure 1).

The complete nucleotide (nt) sequences (50) and partial sequences (9) of plasmids representing each RFLP profile were obtained (Figure 1). This pool includes complete sequences of five plasmids described in our previous studies: pLIS1 (GenBank: NZ_MH382833.1) [20], pLIS3 (NZ_MT912503.1) [24] and pLIS4-pLIS6 (NZ_MW124301.1; NZ_CP063072.1; NZ_MW124302.1) [23], as well as two plasmids that are identical to the previously sequenced *L. monocytogenes* plasmids pLmA144 (KU513859.1) (residing in a strain responsible for a human outbreak in Italy in 2008) and pMF4545 (CP025444.1) [25,26]. The sequences of 48 novel and unique replicons were obtained and named pLIS2, pLIS7-pLIS44, pLIS46-pLIS52 and pLIS54-pLIS55 (plasmid numbers do not refer to the RFLP groups) (Figure 1, Table S1—including the affiliation of the individual plasmids to RFLP groups).

### 2.2. A Dataset of Fully Sequenced Listeria spp. Plasmids

The obtained sequence data were combined with complete plasmid sequences available in the GenBank database (as of 6 December 2020): 56 of *L. monocytogenes*, three of *L. innocua*, 1 of *L. grayi*. A few other sequences (deposited as “complete plasmid sequences”) were excluded from the dataset because they either appeared to be partial sequences or large fragments of chromosomal DNA (Table S2). As a result, a total of 113 unique complete plasmid sequences were further analyzed (their characteristics and GenBank accession numbers are presented in Table S2).

Interestingly, all of the replicons referred to as large plasmids (106 sequences, ranging in size from 15 kb to 153 kb) have related replication systems (REP) encoding a RepA replication initiation protein. These RepAs, containing the helical PriCT_1 domain (PFAM: PF08708) found in the C-termini of primases, show sequence similarity to the replication initiation protein of the well-studied theta-replicating plasmid pAMβ1 of *Enterococcus faecalis*, representing the Inc18 group of plasmids [12,27].

The far less abundant smaller plasmids (7 sequences, <10 kb), are more divergent. Most contain REPs typical for rolling-circle replicating (RCR) plasmids. pDB2011 (KC456362.1) and pIP823 (U40997.1) belong to the pUB110/pC194 family of broad-host-range plasmids [6,22], while pLIS3, pLmN12-0935 (NZ_CP038643.1) and p18-04540 (NZ_CP063384.1) (three highly similar plasmids), carry a different RCR system, also found in plasmids from several gram-positive bacteria [8,24,28]. No known replication systems were identified in the sequences of plasmids pLIS55 (MZ151539) and pAUSMDU00000235 (NZ_CP045971.1).

### 2.3. Conserved Backbone and Structure of the repA-Family Plasmids

The plasmidome of *Listeria* spp. is dominated by a large group of replicons, designated in this study as *repA*-family plasmids. Comparative analysis revealed that the conserved backbone of the vast majority (103/106) of these replicons consists of three components, placed in a conserved order: (i) the *repA* gene of the REP module, (ii) two genes probably involved in active partitioning of plasmid molecules (PAR), and (iii) two or three genes encoding putative subunits of a Y-family DNA polymerase (POL). The plasmids also contain numerous transposable elments (TEs), which have influenced the structure of these replicons.

#### 2.3.1. Replication Systems

**Figure 2:**
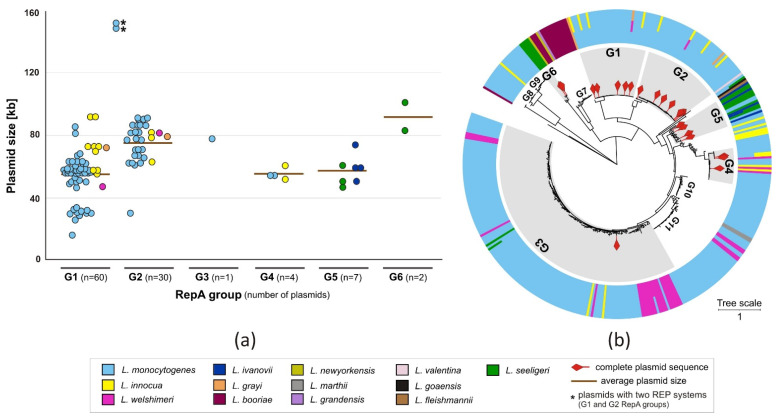
Diversity of the *Listeria* spp. *repA*-family plasmids. (**a**) Size distribution of the *repA*-family plasmids analyzed in this study, grouped according to the RepA phylogenetic clusters. G1–G6 indicate RepA groups. (**b**) Phylogenetic tree of related RepA proteins found in *Listeria* spp. genomes (GenBank); the gray areas indicate proteins belonging to the G1–G6 RepA groups, while the outer ring presents *Listeria* species encoding the RepA proteins. Additional main phylogenetic RepA groups, for which no complete *Listeria* spp. plasmid sequences are yet known, were named G7–G11.

Most of the analyzed *Listeria* spp. plasmids can be categorized into two major RepA phylogenetic groups (G1 and G2), distinguished in previous studies [12,15,16] (Figure S1). The majority of the plasmids occur in *L. monocytogenes* and *L. innocua* strains (Figure 2a). Two plasmids, pN1-011A (149 kb; NC_022045.1) and pCFSAN021445 (152 kb; NZ_CP022021.1), each carry two separate replication systems (G1 and G2), proving that they are composite molecules, probably generated by recombination events between two individual replicons.

The RepA proteins of several plasmids sequenced in this study fall into three additional phylogenetic groups (named G4–G6). Plasmids of the group G4 were described in two recent studies [15,29]—they rarely occur, but encode a putative sanitizer resistance module. Plasmids classified within groups G1, G4 and G5 are on average smaller than those in the other groups. The G5 group is the most divergent, and two plasmids from this group, pLIS46 (MZ147617) and pLIS47 (MZ147618), were harbored by one *L. ivanovii* strain (Sr19), suggesting that they are compatible and can stably coexist in the same cell.

To obtain a more comprehensive picture of the diversity and distribution of RepA proteins in the genus *Listeria*, a PSI-Blast search of the GenBank protein database (as of 24 April 2021) was performed. This analysis resulted in the identification of 418 unique complete RepA proteins from 13 *Listeria* species (phylogenetic tree presented in Figure 2b; a more detailed description of the dataset is presented in Figure S2). To estimate the prevalence of RepA proteins, the number of sequenced *Listeria* spp. strains encoding specific RepA proteins was retrieved from the NCBI database and shown in Figure S2. RepA proteins belonging to G1 and G2 groups were the most commonly found in the sequenced strains. However, it should be noted that most sequenced strains available in the GenBank database belong to *L. monocytogenes* species. Interestingly, over 90% of the *Listeria* strains possessing a RepA protein encoded one of the eight most common variants, with the dominant variant (WP_003728500.1—G1) found in over 6000 strains (Figure S2), and in 48 of the completely sequenced plasmids. Plasmids encoding RepA proteins were found in different *Listeria* species, although some host preferences could be observed, e.g., RepA groups G1 and G2 were not common in *L. seeligeri* and *L. ivanovii* strains, while members of G6 were detected exclusively in the former (Figure 2b).

Interestingly, seven of the *repA*-family plasmids—pLIS2 (MW934262; G1), pLIS9 (MZ043156; G1), pLIS10 (MZ043158, G1), pLIS35 (MZ127844, G1), pLIS37 (MZ127846, G1), pCFSAN010068_01 (NZ_CP014251.1, G1) and pLI100 (NC_003383.1, G2)—carry additional (1 or 2) complete or partial REP modules, unrelated to the *repA* family, or PAR modules located in separate regions of the plasmid genome. Such intra-replicon gene transfer may be associated with transposition and recombination events, since the aforementioned genetic modules are often part of putative composite transposons, flanked by insertion sequences (ISs). All but one of the transferred REP modules (one of the additional REP present in pLI100) are related to each other and show sequence similarities to many *Lactococcus* spp. plasmids. The REP of pLI100 is similar to those of *Listeria* RCR plasmids pDB2011 and pIP823. Such natural bi-replicon plasmids may potentially have the properties of shuttle replicons and extend the host range.

There have been few studies on the host range of large *Listeria* plasmids, and these are usually limited to species within this genus or do not involve plasmid DNA sequencing, e.g., [21]. To test the host range of the analyzed *repA*-family plasmids, a series of mobilizable shuttle plasmids was constructed in *Escherichia coli*. These were composed of (i) vector pDKE2 (contains the R6K origin of replication that is functional exclusively in *E. coli* strains encoding R6K replication initiation protein Pi *in trans*) and (ii) the REP + PAR or REP + PAR + POL modules (Figure S3) of five *Listeria* plasmids (pLIS36—MZ127845, pLIS1, pLIS26—MZ090006, pLIS6, pLIS50—MZ151535) representing the G1, G2, G4 and G5 RepA phylogenetic groups, respectively (Figure S1).

The constructed plasmids were capable of replication in all tested *Listeria* species (*L. monocytogenes*, *L. seeligeri*, *L. ivanovii*, *L. innocua*, *L. grayi*) and *B. subtilis*. With *Staphylococcus aureus*, numerous transconjugants were obtained, but autonomous forms of the introduced plasmids were observed only for the G2 and G4 REP systems. None of the tested plasmids was able to replicate in *E. coli* DH5α (*Gammaproteobacteria*) or *Paracoccus pantotrophus* KL100 (*Alphaproteobacteria*). These observations indicate that the host range of these *repA*-family plasmids may be limited to gram-positive bacteria.

#### 2.3.2. Stable Maintenance Systems

The observed *repA* genes are associated and adjacent with putative *parAB* operons encoding partitioning systems (PAR) that promote stable maintenance of bacterial plasmids. The PAR is responsible for active segregation of newly replicated plasmid copies into daughter cells during cell division, although the putative PAR systems present in *Listeria* plasmids were not yet functionally tested.

**Figure 3:**
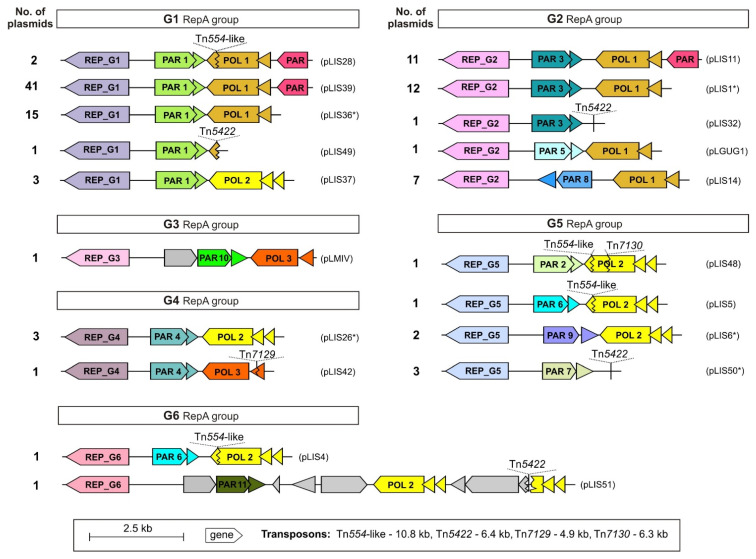
Genetic organization of the conserved backbone of *Listeria repA*-family plasmids, composed of replication (REP), partitioning (PAR) and Y-family polymerase (POL) genetic modules. Number of plasmids with similar genetic organization of backbones is indicated on the left while names of example plasmids are presented on the right. Transposon insertion sites are marked. Stars indicate plasmids whose REP systems were subjected to host range analysis. Phylogenetic trees of the RepA, ParA and UvrX protein sequences, supporting their classification into individual groups, are presented in Figures S1, S4 and S5.

The PAR systems of *Listeria* plasmids (classified to group Ib) are related, and can be roughly divided into 11 phylogenetic groups (Figure S4). They mostly occur together with specific RepA types (e.g., G1, G4), but in some cases (e.g., G2, G6) similar *repA* genes are associated with PAR systems representing different phylogenetic groups and vice versa, i.e., related PAR systems can be associated with distinct RepA proteins. For example, plasmids pLIS4 and pLIS5 encode highly similar ParA and ParB proteins (95% and 87% amino acid (aa) identity, respectively), while their RepA proteins are more divergent (41% aa identity, groups G6 and G5) (Figure 3). This reflects both the modular structure of *Listeria* spp. plasmids and recombinational gene exchange occurring between different *repA*-family replicons. As many as 54 plasmids encode an additional putative ParA-family protein (100% aa sequence identity), located close to the conserved plasmid backbone (PAR; Figure 3; G1 and G2 RepA groups). PAR systems were not found in any of the small *Listeria* RCR plasmids.

Many *repA*-family plasmids (but none of the small *Listeria* RCR plasmids) contain additional stabilization modules, which act by a mechanism involving post-segregational killing of plasmid-less cells. Such systems are usually two-gene operons, encoding a stable toxic protein and a labile antitoxin that neutralizes the toxin’s activity [30].

The most common of these stabilization modules in *Listeria* plasmids are type II toxin-antitoxin (TA) systems, representing two TA families: (i) *mazEF*/*pemIK*—occurring in 49 replicons (47 of these systems are highly similar) and (ii) *relBE*/*parDE*—two such TA *loci* are present in a total of eight plasmids. In addition, putative toxins of type III TA systems were identified in 24 plasmids (Table S2). Plasmids belonging to the G1 RepA group mostly encode type II TA systems, although type III and other putative TA systems are also present. Type III systems are more common in plasmids from the G2 group (Table S2).

None of the TA modules has been experimentally analyzed so far, although a few studies have been performed on chromosomally encoded TAs of *L. monocytogenes* strains [31,32,33].

#### 2.3.3. Y-Family DNA Polymerase Modules

Intriguingly, most of the analyzed *repA* plasmids contain 2 or 3 conserved genes located near the PAR modules (POL module) (Figure 3), which encode putative UvrX and YolD subunits of the Y-family DNA polymerase—possibly equivalents of UmuC and UmuD from gram-negative bacteria [34,35]. Y-family polymerases act as DNA repair systems, which are activated in response to DNA damage (e.g., caused by UV radiation) and specialize in translesion DNA synthesis, bypassing damaged bases that would otherwise block the replication forks [36]. The activity of these polymerases may stimulate spontaneous deletions during DNA repair [37], and could therefore contribute to variation and adaptation of both plasmid and host genes [12].

The genes encoding Y-family polymerases are components of the global SOS system, and their expression may be regulated by the transcriptional repressor LexA [34,35]. Sequences similar to *L. monocytogenes* SOS operator sequences (LexA-boxes) [34,38] were identified upstream of the POL modules in most of the analyzed plasmids (data not shown).

The larger UvrX subunits display a high level of aa sequence conservation (62–100% aa sequence identity) and form three main phylogenetic groups (POL1-POL3; Figure 3 and Figure S5). The polymerase module of the POL2 group contains two putative *yolD* genes. One plasmid (pLIS51; MZ151536) carries an additional copy of these genes, located far from the REP module (Figure 3).

In six of the *repA* plasmids, the transposition of TEs has caused disruption of the gene encoding one of the polymerase subunits (Figure 3). In some of those cases (e.g., pLIS28, pLIS4) transposition caused shortening of the uvrX gene by only about 30 bp; it is possible that the proteins lacking some C-terminal aa are still functional. Interestingly, in five cases (pLIS4, pLIS5, pLIS28, pLIS48, pLIS54) the inserted element was a Tn*554*-like transposon that also harbors a homologous *uvrX* gene. Related UvrX proteins are also commonly encoded by the chromosomes of *Listeria* spp. (Figure S5).

#### 2.3.4. Conserved Structure of the *repA*-Family Plasmids

**Figure 4:**
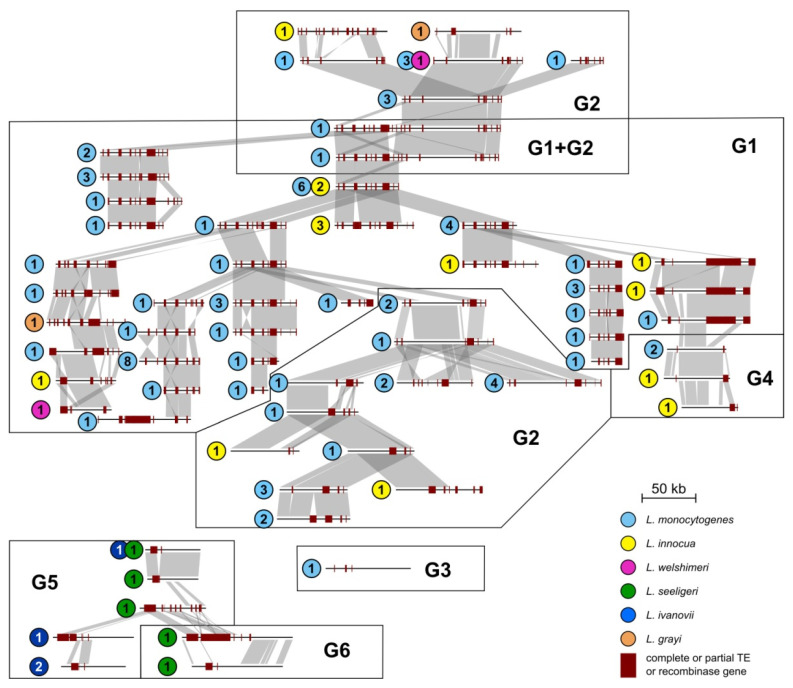
Conserved structure of *Listeria repA*-family plasmids analyzed in this study. Plasmids are grouped within RepA phylogenetic clusters (G1–G6). The sequences were aligned and visualized using EasyFig. Regions of size > 1 kb and nt sequence identity > 90% are connected by gray boxes. Plasmids differing only by point mutations and small indels (of <1 kb) are grouped together and represented by one plasmid of the largest size, with circled figures indicating the number of plasmids included. Inserted TEs (complete and partial) as well as recombinase genes, which may mediate recombination events, are indicated by brown boxes.

Comparative analysis revealed that sequence conservation among the *repA*-family plasmids is not limited to the core REP, PAR and POL modules. Many plasmids have similar structures and share numerous highly conserved DNA segments (Figure 4), as was observed in previous studies [12,14,15,16,17]. The pan-genome of these plasmids consists of around 900 genes, which is a relatively small number considering the fact that these large plasmids carry about 70 genes on average (range: 16–169). The structure and gene content of the vast majority of *Listeria repA* plasmids is highly conserved, and several of the plasmids differ only by point mutations or the presence of small indels of <1 kb in size (Table S2). Sequence conservation between plasmids of different RepA lineages is shown in Figure 4 (a more detailed version, including plasmid names, is available in Figure S6).

In most cases, RepA clustering is associated with a similar gene content (Figure 4; also visualized by a protein-based similarity network of *Listeria* plasmids in Figure S7). Most of these plasmids contain numerous complete and partial TEs (including insertion sequences, mostly of the IS*3*, IS*6* and IS*21* families) and recombinase genes, with an average of around nine such elements and genes per plasmid. The highest number (24) was found in plasmid pCFSAN021445. In many cases, DNA regions of high nucleotide sequence similarity shared by different plasmids are bordered by TEs (Figure 4 and Figure S6), suggesting that these elements may play an important role in the evolution of *Listeria* spp. plasmids by mediating diverse recombinational rearrangements.

### 2.4. Conjugative Transfer Modules of Listeria spp. Plasmids

DNA transfer enables bacteria to acquire new traits. To our knowledge, conjugative transfer of only three of the sequenced *Listeria* plasmids has been experimentally confirmed: pLIS1 [20], pLM1686 (NZ_MK134858.1) [39,40] and a small mobilizable plasmid pIP823 [6]. In addition, it was demonstrated that a plasmid vector encoding a relaxase (an enzyme necessary for the initiation of plasmid conjugal transfer) from pLIS3 could be mobilized between *E. coli* strains in the presence of the helper conjugational system of plasmid RP4 [24]. Several approaches to transfer other plasmids in biparental mating experiments failed, which suggests that the tested replicons are either not self-transmissible or require specific conditions for efficient transfer [7,13,20,23,41] (indicated in Table S2).

Based on in silico predictions, it seems probable that 24 of the analyzed *Listeria* *repA* plasmids contain complete transfer modules (TRA) encoding a MobL-family relaxase [42], cell wall hydrolase, type IV secretion system (T4SS) proteins, a putative toprim domain-containing protein, proteins similar to pilus assembly complex components, surface adhesion proteins, plus many other hypothetical proteins of unknown function (Figures S8 and S9). The predicted TRA modules of plasmids pLIS1 and pLM1686, which were successfully conjugatively transferred between *Listeria* strains, are approx. 25–30 kb-long and are present in 11 and nine plasmids, respectively.

Interestingly, over half of the *repA*-family plasmids from group G2 and all of the G4 group contain complete predicted TRA modules, while plasmids from the G1, G3 and G5 groups seem to carry only partial modules. However, any plasmid carrying a relaxase gene can still potentially be mobilized for conjugative transfer when provided with a functional TRA module *in trans*, for example by another plasmid present in the same cell, or by an integrative conjugative element (ICE) integrated within the chromosome. Most of the small *Listeria* plasmids have the potential to be mobilized for transfer, except two replicons, pAUSMDU00000235 and pLIS55, that do not encode predicted relaxases.

Most *Listeria* plasmids (92) encode related MobL-family relaxases [42], which cluster in 5 main phylogenetic groups (Figure S9). Six plasmids encode multiple relaxases—either (i) two MobL family proteins (pN1-011A, pCFSAN021445), (ii) a MobL and MobL/MobA relaxase (pLIS35), or (iii) two MobL family relaxases and one additional relaxase (pLIS2, pLIS9, pLIS37) encoded within a putative composite transposon (high aa sequence similarity to relaxases of many *Enterococcus* spp. and *Lactococcus* spp. plasmids). In addition, three plasmids (pLIS47, pLIS50, pLIS52—MZ151537) encode a predicted partial TRA system consisting of a gene coding for a replication-relaxation family protein (unrelated to the MobL family) adjacent to genes for T4SS proteins and a peptidase.

### 2.5. Adaptive Genes of Listeria spp. Plasmids

**Figure 5:**
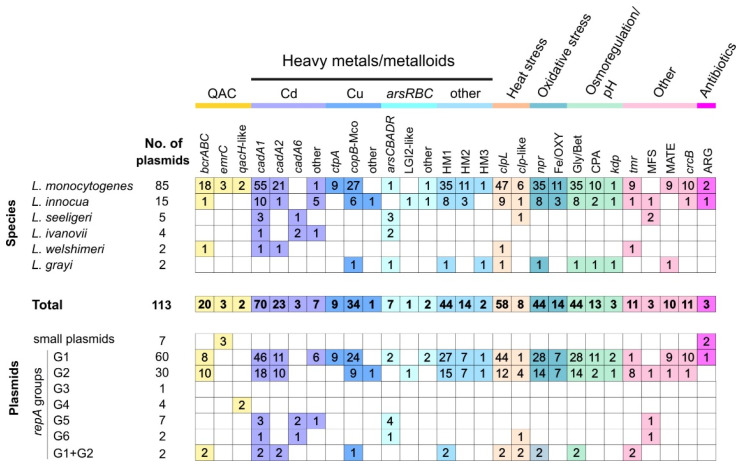
Distribution of selected plasmid-encoded genes potentially involved in stress responses, grouped by *Listeria* species (**upper panel**) or by plasmid replication system group (**lower panel**). Numbers in boxes represent the number of plasmids harboring specific genes. Abbreviations are as follows: QAC—quaternary ammonium compound, Cd—cadmium, Cu—copper, As—arsenic, LGI2-like—the arsenic resistance operon similar to that in genomic island LGI2, HM1-HM3—heavy metal ATPases, Gly/Bet—glycine/betaine ABC transporter, Fe/OXY—proteins putatively involved in iron export or the oxidative stress response, CPA—cation/proton antiporter, MFS—major facilitator superfamily transporters, MATE—multidrug and toxic compound extrusion family pumps, ARG—antibiotic resistance genes. Frame-shifted genes were included when present.

*Listeria* spp. strains can be subjected to diverse environmental stressors; during food production processing, they are often exposed to antimicrobials, high salinity, acidity and non-optimal temperatures. The gene content of the *Listeria* spp. plasmids was analyzed in order to identify genes possibly involved in the response to stress factors, which may contribute to the adaptation of *Listeria* spp. to diverse and changeable environments. Many of the identified modules are shared among plasmids from various *Listeria* species, as well as with bacteria of other genera, suggesting their horizontal transmission. Many of these modules occur within putative composite and non-composite transposons. A summary of this analysis is presented in Figure 5, and the distribution of the identified genes in individual plasmids is presented in Table S2.

#### 2.5.1. Heavy Metal and Metalloid Resistance

Heavy metal resistance determinants are common components of *Listeria* spp. plasmids. The majority of the plasmids (95/113) encode genes conferring resistance to cadmium. So far, seven functional cadmium efflux system genetic variants (*cadA1C1*-*cadA7C7*) have been identified in *Listeria* spp. [9,23,43,44,45,46,47], although only three of them (*cadA1C1*, *cadA2C2* and *cadA6C6*) were carried by plasmids (by 70, 23 and three plasmids, respectively; Table S2). The composite plasmids pN1-011A and pCFSAN021445 carry both the *cadA1C1* and *cadA2C2* variants. Six of the *cadA1C1*-containing plasmids also encode a CadD-family protein, which may potentially facilitate the export of cadmium ions [48].

Interestingly, even though plasmid pLIS48 (MZ147619) contains a putative novel *cadAC* module, the strains carrying pLIS48 or pLIS48-like plasmids were not resistant to cadmium—so either the genes are non-functional, or they confer resistance to other factors [23].

Most of the resistance determinants were harbored by non-composite transposons Tn*5422* (*cadA1C1*), Tn*6870* (*cadA6C6*) or Tn*7130* (putative *cadAC* cassette in pLIS48), or a composite transposon Tn*6869* (pLIS4), which also encodes a set of proteins involved in carbohydrate transport and metabolism [10,23]. The *cadA2C2* cassette is adjacent to serine recombinase and transposase genes, suggesting that it could also be an integral part of a mobile element.

Arsenic resistance in *Listeria* spp. is usually determined by the presence of chromosomally inserted genomic island LGI2 (*arsR1D2R2A2B1B2* and upstream *arsA1D1* cassette) or a Tn*554*-like transposon (*arsCBADR*) [44,46]. These *loci* were linked with plasmids in only a few cases: (i) a Tn*554*-like transposon was found within five plasmids, inserted near the PAR modules (Figure 3), (ii) a partial sequence (*arsADR*) was identified in two plasmids, and (iii) an *arsR1D2R2A2B1B2* operon (flanked by TEs), 99% identical to the locus within island LGI2, was detected in one plasmid (pLI100) (Table S2). Two plasmids (pLIS24 and pLIS36) carry another 3-gene cluster, *arsRBC*, highly similar (99% nt identity) to operons occurring in many *S. aureus* plasmids and chromosomes, which was shown to confer arsenic resistance in *S. aureus* and *B. subtilis* [49]. The strains harboring pLIS24 and pLIS36 were capable of growth in the presence of (meta)arsenite at high concentration (500 µg/mL), which suggests the functionality of this plasmid resistance locus in *Listeria*. Interestingly, most (8/10) plasmids harboring the aforementioned arsenic resistance determinants were isolated from species other than *L. monocytogenes* (Figure 5).

Numerous *Listeria* spp. plasmids (44/113) harbor genes that are likely to be involved in copper detoxification. A copper transporter system gene *ctpA* was identified in a plasmid of *L. monocytogenes* DRDC8 (U15554.3), and is part of a 10-gene cluster involved in copper homeostasis and virulence [50,51,52]. This cluster is present in nine complete *L. monocytogenes* plasmids, of which eight are highly similar replicons (Table S2).

A complete putative copper-resistance cluster consisting of genes for a CopB-family copper-translocating P-type ATPase, multi-copper oxidase Mco and a YdhK-family protein of unknown function, is present in two plasmids, pLIS28 and pLIS54 (Table S2). Two variants of the CopB are encoded by these plasmids, differing by a 17 aa duplication. Genes encoding the shorter or longer variants of CopB are present in 32 plasmids, but the Mco gene is truncated by a transposon insertion and subsequent recombination events. The *copB-mco* operon is highly similar (99% nt identity) to those found in several *S. aureus* chromosomes and plasmids, including plasmid SAP078A (NC_019009).

The *copB-mco* operon of plasmid SAP078A confers copper hypertolerance in *S. aureus* strains and enhances bacterial survival inside macrophages [53]. Whether this operon performs a similar function in *L. monocytogenes* strains remains unknown. Interestingly, expression of the *mco* gene of some *Listeria* plasmids was shown to be upregulated during growth under salt and acid stress conditions [16,54]. In plasmids pLIS28 and pLIS54, the operon is located between two serine recombinase genes (this resistance *locus* is also adjacent to recombinase genes in most *S. aureus* sequences, including SAP078A).

Plasmid pLIS41 (MZ147614) encodes another putative copper detoxification cluster—a copper translocating P-type ATPase and a CopY-family repressor. This cluster shares 99–98% nt sequence identity with several *Lactobacillus* spp. and *Lacticaseibacillus* spp. plasmid and chromosome sequences. Ten other plasmids harbor the *copY* gene and a partial ATPase gene.

The analyzed plasmids also carry genes for three other putative heavy metal translocating P-type ATPase types, but the substrates of these proteins remain unknown. One of these proteins is encoded by 44 plasmids, of which 14 also carry genes encoding a second type of P-type ATPase (Table S2). The third type is encoded by only two plasmids—pLGUG1 (NC_014496) and pNH1 (NZ_MH277333) [7,12]. In pLGUG1 the ATPase gene forms part of a putative IS*1216*-driven (IS*6* family) composite transposon, flanked by directly repeated sequences (DRs)—scars of a transposition event. The genes encoding ATPases of types two and three display high similarity (98–99% nt identity) to sequences present in many *Enterococcus* spp. strains.

#### 2.5.2. Sanitizer Resistance

Resistance to quaternary ammonium compounds (QACs), which are commonly used as sanitizers in food-processing environments, is often observed in *Listeria* spp. strains [20,21,55,56]. Several different QAC efflux pumps have been identified in *Listeria* spp., two of which are commonly associated with plasmids—*bcrABC* [9] and *emrC* [8].

The *bcrABC* cassette is present in 20 plasmids (Table S2). In many cases, this cassette is associated with *cadA2C2* and a triphenylmethane reductase gene *tmr* (present in some *Listeria* plasmids), and may be part of a putative composite transposon, although no flanking DRs were identified [9]. In plasmids pLIS33 and pCFSAN022990 [57], the cassette is part of another putative IS*1216*-driven composite transposon, flanked by DRs. The *emrC* gene was only detected in three nearly identical small plasmids, which differ by point mutations (Table S2).

Additionally, plasmids pLIS12 and pLIS26 encode a transcriptional regulator and a SMR protein, closely related (93% aa identity) to the *qacH* QAC resistance efflux pumps of Tn*6188* [58]. Strains harboring this gene cassette were resistant to benzalkonium chloride (BC), but further experiments are required to confirm its specific function. Interestingly, the cassette is bordered by 37-bp-long inverted repeats (with 32/37 bp identity to those of the Tn*3*-family transposon Tn*7129* present in 8 *Listeria* plasmids) and flanked by 5-bp-long DRs. This is a structure typical for non-autonomous mobile insertion cassettes (MIC). Closely related MICs (99% nt identity) are also present in plasmids from *Enterococcus faecalis* (LR962259.1) and *Clostridioides difficile* (LR595854.1) strains. 

#### 2.5.3. Antibiotic Resistance

Surprisingly, very few *Listeria* spp. plasmids have been connected with antibiotic resistance [7,22,59,60,61]. Two small plasmids, pIP823 and pDB2011, were shown to confer resistance to trimethoprim (*drfD*) [6,22,60]. The latter replicon additionally determines resistance to spectinomycin (*spc*), and carries a probably non-functional erythromycin resistance gene (*erm(A)*) [22]. These two broad-host-range plasmids show little similarity to other *Listeria* plasmids, and were probably recently acquired from *Staphylococcus* spp., as has also been suggested in the case of two multidrug resistance plasmids pIP811 and pWDB100 (37 kb and 39 kb; undetermined sequences) [59,61]. Sequences similar to plasmids pIP823 and pDB2011 were identified in only a few *Listeria* strains (WGS data; GenBank), indicating that they are probably a recent acquisition by this genus.

A large multi-resistance plasmid, pNH1, was recently identified in a *L. monocytogenes* strain isolated from frozen food in China [7]. The backbone of this plasmid shares high sequence similarity with many *Listeria* plasmids. The main antibiotic resistance gene cluster, conferring resistance to tetracycline (*tet(S)*), lincosamides (*lnu(B)*), PLS_A_ (*lsa(E)*), spectinomycin (*spw*), streptomycin (*aadE*), macrolides, lincosamides and streptogramin B (*erm(B)*), kanamycin and neomycin (*aphA3*), and chloramphenicol (*catA8*), was probably introduced on a large IS*1216*-driven composite transposon (23 kb), flanked by 8-bp-long DRs.

Plasmid pNH1 contains multiple copies of IS*1216*, which can result in the generation of various composite transposons, one of which was shown to be functional (Tn*6659*) [7]. Plasmid pNH1 also carries a trimethoprim resistance gene (*drfG)* introduced by a putative TE (3 kb), which is also present in numerous *Enterococcus* spp. and *Staphylococcus* spp. plasmid and chromosome sequences (99–100% nt identity). This element was previously identified as a component of transposon Tn*6198* [62].

Among the plasmids sequenced in this study only pLIS35 harbors two putative aminoglycoside phosphotransferase family proteins. However, none of the seven *L. innocua* and *L. welshimeri* strains harboring pLIS35 and pLIS35-like plasmids were resistant to any of the nine aminoglycosides when tested in antimicrobial susceptibility assays.

#### 2.5.4. Heat Stress

An ATP-dependent protease ClpL from plasmid pLM58 was shown to be involved in increased heat resistance of *L. monocytogenes* strains [13]. The ClpL gene is present in more than half (58/113) of the analyzed *Listeria* plasmids. Interestingly, this gene was also found to be upregulated in response to low pH [16,54]. Genes encoding two other shorter proteins belonging to the ClpA/ClpB family, are present in eight plasmids (Table S2); they may also be potentially involved in a heat-shock response, but this has not been experimentally tested.

#### 2.5.5. Oxidative, Acid and High Salinity Stresses

*Listeria* spp. strains are often exposed to high salinity and low pH conditions in food production facilities and food products. Genes putatively involved in osmotic, acid and oxidative stress responses were identified in *Listeria* plasmids based on similarity to homologs from other bacteria [12], but their functions have not been experimentally verified. Comparisons of wild-type and plasmid-cured strains indicate that some *Listeria* plasmids could play a role in growth and survival under salt, acid and oxidative stress conditions [63], but the underlying mechanisms remain unknown.

A region encoding a heavy metal resistance ATPase, a glycine betaine ABC transport system substrate-binding protein and an oxidoreductase (NADH peroxidase; *npr*) is present in 44 *Listeria* plasmids (Table S2). The latter two genes may play a role in responses to high osmolarity and oxidative stress, and were shown to be upregulated under high salt conditions [16,64,65,66,67]. This DNA segment is flanked by TEs, and a similar region (99% nt identity) is present in an *E. faecalis* plasmid (LR961976.1). In three plasmids (pLIS2, pLIS9, pLIS37), this region forms part of a large IS*Lmo13*-driven composite transposon (31 kb), bordered by DRs. Moreover, several *E. faecalis*, *Weissella* spp. and *Aerococcus* spp. strains harbor homologous genes encoding glycine betaine permease and oxidoreductase (97–99% nt identity).

Another DNA region, encoding a cation/proton antiporter (potential role in maintaining pH), an enolase and CrcB-family fluoride efflux transporters, is present in 11 plasmids, while two plasmids carry only the enolase and antiporter genes (Table S2). A region containing a putative iron export ABC transporter subunit and a protein with a ferritin-like iron-binding domain is present in 14 plasmids (Table S2). It has been suggested that these genes are involved in the oxidative stress response [63]. A highly similar region (99% nt similarity) is present in many *Enterococcus* spp. plasmids and chromosome sequences.

Finally, three plasmids (pLIS54, pLI100, pNH1) from three different *Listeria* species carry a six-gene cluster related to the Kdp-type potassium transport system adjacent to a serine recombinase gene. Homologous systems, present in many bacterial genomes, including *L*. *monocytogenes* chromosomes, are involved in osmotic and pH adaptation [68,69].

#### 2.5.6. Other Genes of Potential Adaptive Value

Eleven *Listeria* plasmids encode a triphenylmethane reductase (*tmr*), which was shown to be involved in toxic dye (e.g., crystal violet) detoxification [70]. In all cases, the *tmr* gene is located close to the *bcrABC* cassette. Triphenylmethane dyes have industrial applications, and may be released into the natural environment. They are also illegally used in fish farming to prevent infections in fish [71]. All strains for which complete plasmid sequences were obtained were tested for crystal violet detoxification, and only those with plasmids encoding the *tmr* gene (pLIS1, pLIS8, pLIS16, pLIS29; Table S2) were capable of growth at high concentrations of crystal violet. However, strains harboring pLIS35 and pLIS35-like plasmids, which also carry the *tmr* gene, were, for unknown reasons, incapable of crystal violet detoxification.

Other genes potentially involved in the efflux of toxic compounds include two distinct MATE (multidrug and toxic compound extrusion) family efflux pumps. One is present in plGUG1 and the other in nine closely related plasmids (Table S2) where the MATE transporter is adjacent to the *ctpA* gene, a copper resistance determinant.

In addition, plasmids pLIS4 and pLIS5 carry a gene encoding a MFS (major facilitator superfamily) transporter and eight nearby genes putatively involved in carbohydrate transport and metabolism. Plasmid pLIS34 encodes a bi-functional aspartate transaminase/aspartate 4-decarboxylase and aspartate-alanine antiporter that are highly similar (99% aa identity) to proteins found in multiple *Enterococcus* spp. strains, and form part of a putative IS*1062*-driven composite transposon flanked by DRs.

Finally, the small plasmid pAUSMDU00000235 (2.7 kb) encodes a putative bacteriocin and bacteriocin immunity protein. A plasmid (2.9 kb) producing a functional bacteriocin was previously reported in a *L. innocua* strain [72]; the bacteriocin and immunity protein region was sequenced (AF330821), and it shares high nt similarity with the related region of pAUSMDU00000235.

#### 2.5.7. Putative Virulence Factors

Most *Listeria* plasmids do not seem to enhance virulence. In fact, a survey of plasmids in the NCBI database suggested that extrachromosomal replicons are markedly less prevalent in clinical *L. monocytogenes* strains than in food-associated or environmental strains [15]. The first potential virulence plasmid described in *L. monocytogenes* was pLMIV—the only known *Listeria* plasmid that encodes four internalin-like proteins. However, the invasion efficacy in human epithelial cells of the pLMIV-cured strain did not differ from the parental strain [41]. Studies on the small pLMST6-like plasmids suggest that they may contribute to increased virulence of *L. monocytogenes* strains, although the mechanism remains unknown [73].

### 2.6. Listeria Plasmids Pangenome Estimation

Global comparative analyses of the *Listeria* spp. plasmid pangenome, in relation to the whole pangenome of *Listeria* spp. as well as to the plasmidome of bacteria of other genera of the type *Firmicutes*, were performed. The main goal was to collect information on: (i) the rate of DNA transfer between *Listeria* spp. plasmids and chromosomes, (ii) the role of plasmids in shaping the pangenome of these bacteria and (iii) the prevalence of related plasmids in other *Firmicutes*.

A total of 893 gene clusters were identified in the complete plasmid sequences of *Listeria* spp. analyzed using the bacterial pangenome analysis pipeline Panaroo (Table S3). Of these, 411 clusters were found only in plasmids sequenced in this study. Apart from transposases, subunits of the Y-family polymerase are among the most prevalent gene clusters found in the analyzed *Listeria* plasmids.

A total of 14,765 gene clusters were identified when the available complete sequences of both chromosomes (244 sequences) and plasmids of *Listeria* spp. were analyzed (Table S4). Only 119 of these gene clusters were found in both the analyzed chromosomes and the plasmids. However, it should be noted that the clusters created by Panaroo include both complete and partial genes, and that while the sequence identity is set to 95%, the Panaroo algorithm allows for clusters below this threshold based on additional context support. Interestingly, the Y-family polymerase gene cluster is also the most commonly found cluster shared between chromosomes and plasmids, being present in over 97% of the analyzed chromosomes and in around 85% of the plasmids.

Almost a quarter of the common gene clusters (present both in chromosomes and plasmids) are part of putative TEs (including genes carried by composite transposons) in some of the plasmids. Another quarter are parts of a prophage region present in one plasmid, pCFSAN008100b, and multiple chromosomes. Around 8% are part of the arsenic operon shared between plasmid pLI100 (where it is flanked by two ISs) and the chromosomal LGI2 genomic islands. Many of the remaining shared genes are located in plasmids in close proximity to genes encoding serine recombinases.

Panaroo identified a total of 73,928 gene clusters in the 5533 complete plasmid sequences from the phylum *Firmicutes* available in GenBank as of 6 December 2020 (Table S5). Only 178 of these clusters are shared between plasmids of *Listeria* and other *Firmicutes*—commonly with *Enterococcus* spp. and *Lactococcus* spp. plasmids. Around half of these plasmid-borne clusters are connected with TEs, while around a quarter are putatively involved in adaptation to stress conditions.

**Figure 6:**
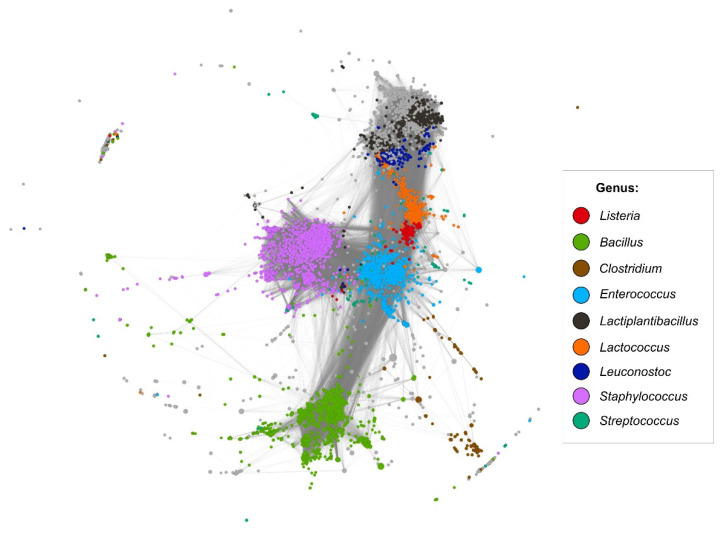
Protein-based similarity network of *Firmicutes* plasmids (sequences available in GenBank). Each node (*n* = 5533) represents a complete plasmid sequence with the size of the node corresponding to the size of the plasmid. Colors indicate the host genus of the most prevalent groups (>2% of the presented plasmids belonging to this genus). Plasmids originating from the remaining hosts are represented in grey. Edges reflect similarity between connected nodes.

A protein-based similarity network (less stringent identity thresholds than the Panaroo analyses) was constructed to visualize similarities between *Listeria* spp. plasmids and those of other *Firmicutes* genera. Most of the *repA*-family plasmids of *Listeria* form a tight cluster of highly related replicons (Figure 6), which correlates with the conserved structure of these replicons (Figure 4). However, the location of the *Listeria* plasmids within the network indicates that they share many genes with plasmids of other *Firmicutes*, which proves their common evolutionary history. In particular, most of them locate between *Lactococcus* and *Enterococcus* plasmids. Of these, the *Enterococcus* ones seem to act as the main bridging hub between *Staphylococcus* and *Bacillus* plasmids, as well as the mosaic cluster at the top of the network. In fact, this cluster is composed mostly of *Leuconostoc* and *Lactiplantibacillus* plasmids but the majority of the grey nodes in that part of the network correspond to plasmids carried by other representatives of closely related genera of the family *Lactobacillaceae*.

## 3. Materials and Methods

### 3.1. Bacterial Strains and Growth Conditions

The 782 analyzed *Listeria* spp. strains, originating from food sources, food production plants and environmental soil and water samples, are listed in Supplementary Materials Table S1. We have previously described 566 of these strains with regard to their resistance profiles and the presence of plasmids: 287 strains from fish, fish products and food-producing factories [24], 155 strains from natural, agricultural and soil samples [23] and 124 strains from other foods and food-processing environments [4,20]. Apart from 12 USA strains, indicated in Table S1, all of the analyzed strains were isolated in Poland between 2001–2017.

Three *Esherichia coli* strains were used for cloning and host range experiments: DH5α [74], DH5α *pir* [75] and β2163 [76]. The following bacterial strains were used in biparental mating experiments: streptomycin resistant *L. monocytogenes* strain 10403S (serotype 1/2a) [77], *L. innocua* PZH 5/04, *L. ivanovii* PZH 7/04, *L. seeligeri* ATCC 25967, *L. grayi* ATCC 25401, *L. welshimeri* ATCC 35897, *Staphylococcus aureus* ATCC 29213, *Bacillus subtilis* 168 [78] and *Paracoccus pantotrophus* KL100 [79].

Unless otherwise specified, *Listeria* spp., *B. subtilis* and *S. aureus* strains were grown in brain-heart infusion (BHI) broth (BioMaxima, Lublin, Poland) or on BHI agar plates (1.5% *w*/*v* agar) at 37 °C. *E. coli*, and *P. pantotrophus* strains were grown in lysogeny broth (LB, BioMaxima) or LB agar plates (LA). When necessary, the media were supplemented with kanamycin (BioMaxima)—50 μg/mL, streptomycin (BioMaxima)—100 μg/mL, erythromycin (Sigma-Aldrich, St. Louis, MO, USA)—2 μg/mL for *Listeria* spp., *B. subtilis*, *S. aureus* and 250 μg/mL for *E. coli*, and diaminopimelic acid—60 μg/mL (DAP) (Sigma-Aldrich). For long-term storage, the isolates were suspended in BHI containing 20% glycerol and frozen at −80 °C.

### 3.2. Plasmid DNA Isolation and Restriction Fragment Length Polymorphism (RFLP) Profile Analysis

Plasmid DNA was isolated from *Listeria* spp. and *B. subtilis* strains, as described previously [23]. In the case of *S. aureus* strains, lysostaphin (A&A Biotechnology, Gdańsk, Poland) was used instead of lysozyme (Sigma-Aldrich). Plasmid DNA for sequencing was isolated using large-scale preparation methods [80], and sequence assembly was verified by RFLP analysis. Purified plasmid DNA was digested with restriction enzymes EcoRI, BamHI or NcoI, according to the manufacturer’s protocol (Thermo Scientific, Waltham, MA, USA). The resulting DNA fragments were separated by electrophoresis in 0.8% agarose gels.

### 3.3. DNA Sequencing

Sequencing of *Listeria* plasmids was performed in the DNA Sequencing and Oligonucleotide Synthesis Laboratory (oligo.pl) at the Institute of Biochemistry and Biophysics, Polish Academy of Sciences. The sequences were obtained using a combination of GridION (Oxford Nanopore Technologies, Oxford, UK) and MiSeq (Illumina, San Diego, CA, USA) sequencing, and assembled with Unicycler [81]. The nucleotide sequences of all plasmids have been deposited in GenBank (NCBI) under the following accession numbers: MW927705; MW934262; MZ043154–MZ043158; MZ065170; MZ089994–MZ090010; MZ127840–MZ127849; MZ147614–MZ147620; MZ151535–MZ151539; and MZ230002 (Supplementary materials Table S2).

### 3.4. Heavy Metal, Metalloid, Benzalkonium Chloride and Crystal Violet Susceptibility

The susceptibility of selected *Listeria* spp. strains to cadmium chloride, sodium(meta)arsenite and BC was determined using an agar dilution method, as described previously [24]. Strains were classified as resistant if they produced confluent growth on plates containing ≥75 μg/mL cadmium chloride, ≥500 μg/mL sodium (meta)arsenite or ≥10 μg/mL BC. Positive and negative control strains were included in all experiments, with inhibitory concentrations that were determined in a previous study [24]. The susceptibility of *Listeria* spp. to crystal violet (CV) was determined using an agar dilution method, as described previously [70], with some modifications. Briefly, several colonies picked from BHI agar plates were suspended in saline solution to obtain a turbidity of 0.5 McFarland units (corresponding to approximately 10^8^ CFU/mL). Spots of 5 µL of each bacterial suspension were applied to BHI plates supplemented with 15 μg/mL CV (Chempur, Piekary Śląskie, Poland). The plates were then incubated at 37 °C for 48 h. Strains were classified as resistant to CV if they produced confluent growth.

### 3.5. Antimicrobial Susceptibility

The resistance of *Listeria* spp. strains harboring pLIS35 and pLIS35-like plasmids (Table S1) to 9 antimicrobial agents was tested using a disc diffusion method, performed according to European Committee on Antimicrobial Susceptibility Testing (EUCAST) guidelines. *L. welshimeri* ATCC 35987 and *L. innocua* PZH 5/04 were used as susceptible control strains. The following antimicrobial discs were used: spectinomycin (10 µg), spectinomycin (25 µg), spectinomycin (100 µg), streptomycin (10 µg), netilmicin (30 µg), tobramycin (10 µg), kanamycin (30 µg), amikacin (30 µg), gentamycin (10 µg) and neomycin (30 µg). Single colonies were selected after 24 h of incubation on BHI agar and suspended in sterile saline solution to obtain a turbidity of 0.5 McFarland units. Mueller-Hinton agar (Thermo Scientific), supplemented with 5% defibrinated horse blood, was inoculated by swabbing in three directions with a sterile cotton swab dipped in the suspension. Antimicrobial discs were then applied, and the plates incubated at 37 °C for 18 ± 2 h, after which inhibition zone diameters were measured. The inhibition zones of the tested strains were compared with those of the control strains.

### 3.6. Shuttle Vector Construction

*E. coli*—*Listeria* spp. shuttle vectors were constructed by cloning replication and stabilization systems from plasmids pLIS1, pLIS6, pLIS26, pLIS36 and pLIS50 into the multicloning site of mobilizable *E. coli* vector pDKE2 [23], containing kanamycin and erythromycin resistance cassettes as selection markers for gram-negative and gram-positive bacteria, respectively. Oligonucleotide primers and restriction enzymes used for cloning, as well as the cloned plasmid fragments, are shown in Supplementary Figure S3. The modules were amplified from purified plasmid DNA by PCR using Phusion High-Fidelity DNA Polymerase (Thermo Scientific), digested with restriction enzymes and ligated with linearized vector DNA using T4 DNA ligase (Thermo Scientific). The ligated DNAs were used to transform chemically competent *E.coli* DH5α *pir* with selection on LA plates containing kanamycin. Recombinant constructs isolated from transformants were verified by restriction enzyme analysis. Isolated plasmid DNA was then introduced into chemically competent DAP auxotrophic *E. coli* β2163 cells, with selection on LA plates containing kanamycin and DAP.

### 3.7. Assessing Host Range of Shuttle Vectors

Biparental mating between donor (*E. coli* β2163 strains harboring constructed shuttle vectors) and recipient strains (*L. monocytogenes* 10403S, *L. innocua* PZH 5/04, *L. ivanovii* PZH 7/04, *L. seeligeri* ATCC 25967, *L. grayi* ATCC 25401, *L. welshimeri* ATCC 35897, *S. aureus* ATCC 29213, *B. subtilis* 168 and *P. pantotrophus* KL100) was used to assess the host range of the constructed plasmids. Overnight cultures of the donor and recipient strains were mixed at a 1:1 ratio, and 100 µL of the mixtures were spread on BHI agar plates containing DAP. After 24 h of incubation at 37 °C, the bacteria were washed off the plates with LB medium, collected by centrifugation and re-suspended in 1 mL of LB. Suitable dilutions were plated on BHI medium supplemented with erythromycin (gram-positive recipients) and incubated at 37 °C for 48 h. In the case of *P. pantotrophus* KL100, kanamycin was used and the plates were incubated at 30 °C. The resulting colonies were streaked onto BHI medium containing erythromycin, as well as in the case of *Listeria* spp. transconjugants, also on ALOA medium (BioMaxima) with erythromycin. The presence of autonomous transferred plasmids in transconjugants was confirmed by plasmid DNA isolation. Transformation of chemically competent cells with purified plasmid DNA (selection on LA plates with kanamycin) was used to test if the constructed vectors were capable of replicating in *E. coli* DH5α (not containing the *pir* gene). No transconjugants were obtained for any of the recipient strains when the donor strain harbored the empty pDKE2 vector.

### 3.8. Bioinformatic Analyses

Automatic annotation of all the complete plasmid sequences obtained in this study (available in the GenBank database) was performed using RAST on the PATRIC platform [82,83], followed by manual refinement in Artemis [84], based on BLAST homology searches performed at the National Center for Biotechnology information (NCBI) website (https://www.ncbi.nlm.nih.gov (accessed on 24 April 2021)) and HHpred (https://toolkit.tuebingen.mpg.de/tools/hhpred (accessed on 24 April 2021)) [85]. Comparative genomics of the plasmids was visualized using EasyFig [86]. Comparative analyses of the TRA systems was performed with Clinker [87]. Novel transposable elements identified in this study were deposited in the ISfinder database [88] and The Transposon Registry [89]. Phylogenetic trees of RepA, ParA, UvrX and MobL proteins were constructed in MEGAX [90], with parameters and accession numbers of the analyzed proteins available in Supplementary Figures S1, S4, S5 and S9, respectively. Visualization of the RepA phylogenetic tree was performed using iTOL [91].

All data used for comparative genomic analyses were downloaded from the NCBI GenBank or RefSeq databases on 6 December 2020. *Listeria* spp. and *Firmicutes* spp. plasmids were recovered from the nucleotide database using the following query terms: *Listeria[ORGANISM] AND plasmid[filter] AND “complete sequence”* and *Firmicutes[ORGANISM] AND plasmid[filter] AND “complete sequence”*, respectively. Plasmids for which both GenBank and RefSeq records were present were filtered, and only the latter was retained for further analysis. Moreover, additional filtering was applied after manual inspection of their content, i.e., based on descriptions including keywords such as: “partial”, “ribosomal”, “lncRNA”, “cloning vector”, “artificial sequence”(Tables S2 and S5). *Listeria* spp. genomes were recovered from the NCBI assembly database using *Listeria[ORGANISM]* as the query. In the case of genome assemblies for which GenBank and RefSeq records were available, only the latter was retained for further analysis.

*Listeria* spp. plasmids identified in this study were compared in three schemes: against other *Listeria* spp. plasmids, against other *Listeria* spp. plasmids and chromosomes, as well as against other *Firmicutes* spp. plasmids present in public repositories. The comparisons were conducted based on Panaroo v1.2.7 pangenome analysis considering the data specific for each scheme [92]. Each analysis was run with the following parameters: *--threshold 0.95 --len_dif_percent 0.95 --clean-mode sensitive --remove-invalid-genes --merge_paralogs --min_trailing_support 1*. The resulting gene groups were further used for COG classification analysis using rspblast v2.10.1 with an e-value of 1 × 10^−5^ as the threshold and considering only the best blast hit *(--max_target_seqs 1 --max_hsps 1*) [93,94]. Protein-based network analysis of *Firmicutes* spp. plasmids employed an all-against-all blastp search of plasmid-encoded proteins. The search was conducted with an e-value of 1 × 10^−10^ and minimum 95% query coverage per HSP (QCOV) and 75% sequence identity (PIDENT) as thresholds. The resulting similarities were parsed with a Python script and the weight of each edge connecting two nodes was calculated as the sum of multiplications of QCOV and PIDENT between all proteins encoded by each plasmid divided by the total number of proteins encoded by a pair of plasmids. The network was laid out in Gephi using the ForceAtlas2 algorithm [95,96].

## 4. Conclusions

This study provides the first large-scale in-depth insight into the structure and genetic composition of plasmids occurring in bacteria of the genus *Listeria*. Plasmids are common in this genus, and they were present in 47% of the 782 tested strains. We analyzed the sequences of 113 plasmids, representing 69 structural variants. These replicons were identified in six *Listeria* species, which enabled us to draw more general conclusions concerning gene flux in this genus.

Intriguingly, we found very low diversity among *Listeria* spp. plasmids. All plasmids considered as theta-replicating, irrespective of their size (15–153 kb), carried closely related REP systems similar to that of *E. faecalis* plasmid pAMβ1 (28 kb) [97]. Most available data concerns *L. monocytogenes* strains isolated from food-related sources. However, our analyses included 256 *Listeria* strains belonging to species other than *L. monocytogenes* (originating from various sources), and all plasmids found within those strains also belonged to the *repA* family, suggesting a more general trend among *Listeria* species. Related plasmids are prevalent in *Firmicutes*, although the possibility that *repA*-family plasmids are in some way favored in *Listeria* spp cannot be excluded. It would be useful to know whether other types of theta-replicating plasmids occurring in gram-positive bacteria can be maintained in *Listeria* spp. We have found two reports of the transfer of natural conjugative plasmids from *E. faecalis* (pAMβ1) and *Streptococcus agalactiae* (pIP501) into *Listeria* spp. recipients [98,99], but in both cases the introduced plasmids encoded RepA proteins of the pAMβ1-type. Interestingly, many pAMβ1 family plasmids found in other *Firmicutes*, including pAMβ1 and pIP501, encode antibiotic resistance genes [27], which are very rare in *Listeria* species.

Another interesting finding concerns the conserved backbone of the *repA* plasmids, which, apart from the replication and partitioning systems, typically includes POL modules, encoding Y-family polymerases. The evolutionary conservation of these genes and their proximity to the REP and PAR modules, strongly suggests that they may play an important role (direct or indirect, e.g., as a component of a plasmid gene regulatory network) in the maintenance of the plasmids. In a few cases, the polymerase genes were disrupted by a transposon, although their absence could be complemented by chromosomally-encoded enzymes, since homologous genes are present in over 97% of sequenced *Listeria* chromosomes. Undoubtedly, understanding the role of the POL modules in the biology of *Listeria* plasmids is an important task for future studies.

The *repA*-family plasmids show a very high degree of structural conservation. Moreover, highly similar plasmids were identified in bacterial strains isolated from different countries in separate years, sources and even from different *Listeria* species. For example, the most prevalent plasmid RFLP group (no. 1—Figure 1; pLIS13), gathering replicons from 102 *L. monocytogenes* strains tested in this study (Table S1), is very closely related to pLM6179-like plasmids isolated from numerous *L. monocytogenes* strains previously identified in many different countries [18,19].

The chromosomes of *Listeria* spp. are characterized by a stable and conserved genetic structure [44,100], which suggests that the phenotypic variation of these bacteria may be largely determined by extrachromosomal replicons. Due to their low diversity, the studied plasmids have a limited impact on the size of the *Listeria* pangenome, but they contain a large reservoir of predicted adaptive genes, which may play a vital role in the response of their host strains to different environmental stressors. It should be emphasized that such genes may potentially impact other phenotypic traits, as was previously observed in the case of some cadmium resistance determinants (*cadAC*), which also influenced virulence and biofilm formation of *L. monocytogenes* strains [45,101].

## Supplementary Materials

The following are available online at www.mdpi.com/article/10.3390/ijms221910320/s1. References [6,7,12,13,19,22,24,25,26,28,40,41,57,73,102,103,104,105,106,107,108,109,110,111,112,113,114] are cited in the supplementary materials.
