# Supplementary material for: Plasmidome of Listeria spp.—The repA-Family Business
Source: Int J Mol Sci. 2021 Sep 25;22(19):10320. doi: 10.3390/ijms221910320 (PMC8508797; doi:10.3390/ijms221910320)
Supplement: Supplementary file 1 [file ijms-22-10320-s001.zip › Figures_S1_to_S9.pdf]

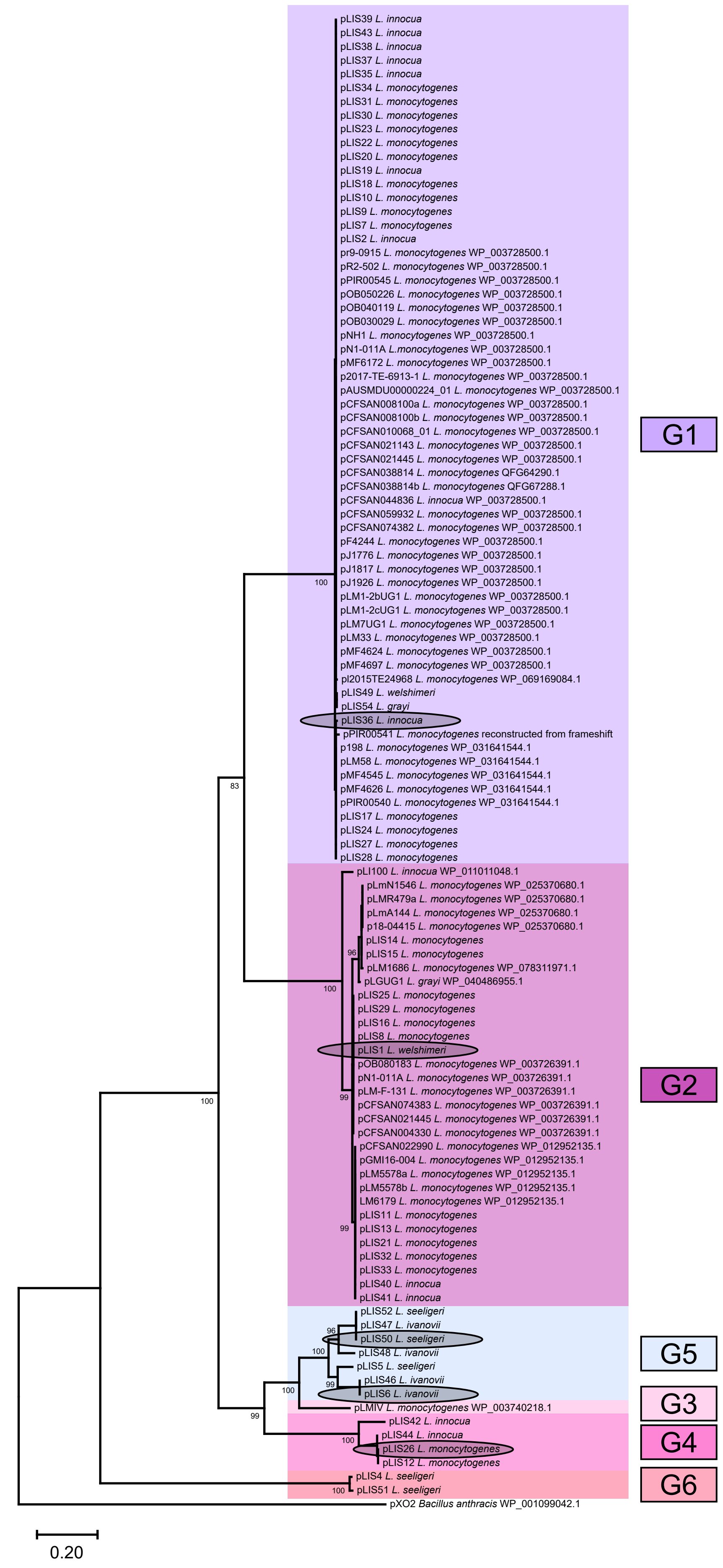

Figure S1. Phylogenetic tree of the RepA proteins encoded by complete large *Listeria* spp. plasmid sequences. RepA from plasmid pXO2 *Bacillus anthracis* (NZ_CP023003.1) was used as an outgroup. The tree was constructed in MEGAX based on 538 amino acid positions (positions with l<95% site coverage were eliminated), using the maximum-likelihood method (Le and Gascuel model). The tree is drawn to scale, with branch lengths representing the number of substitutions per site. Statistical support for the internal nodes was determined by 1000 bootstrap replicates. Plasmids used for shuttle vectors construction are indicated with gray ovals.

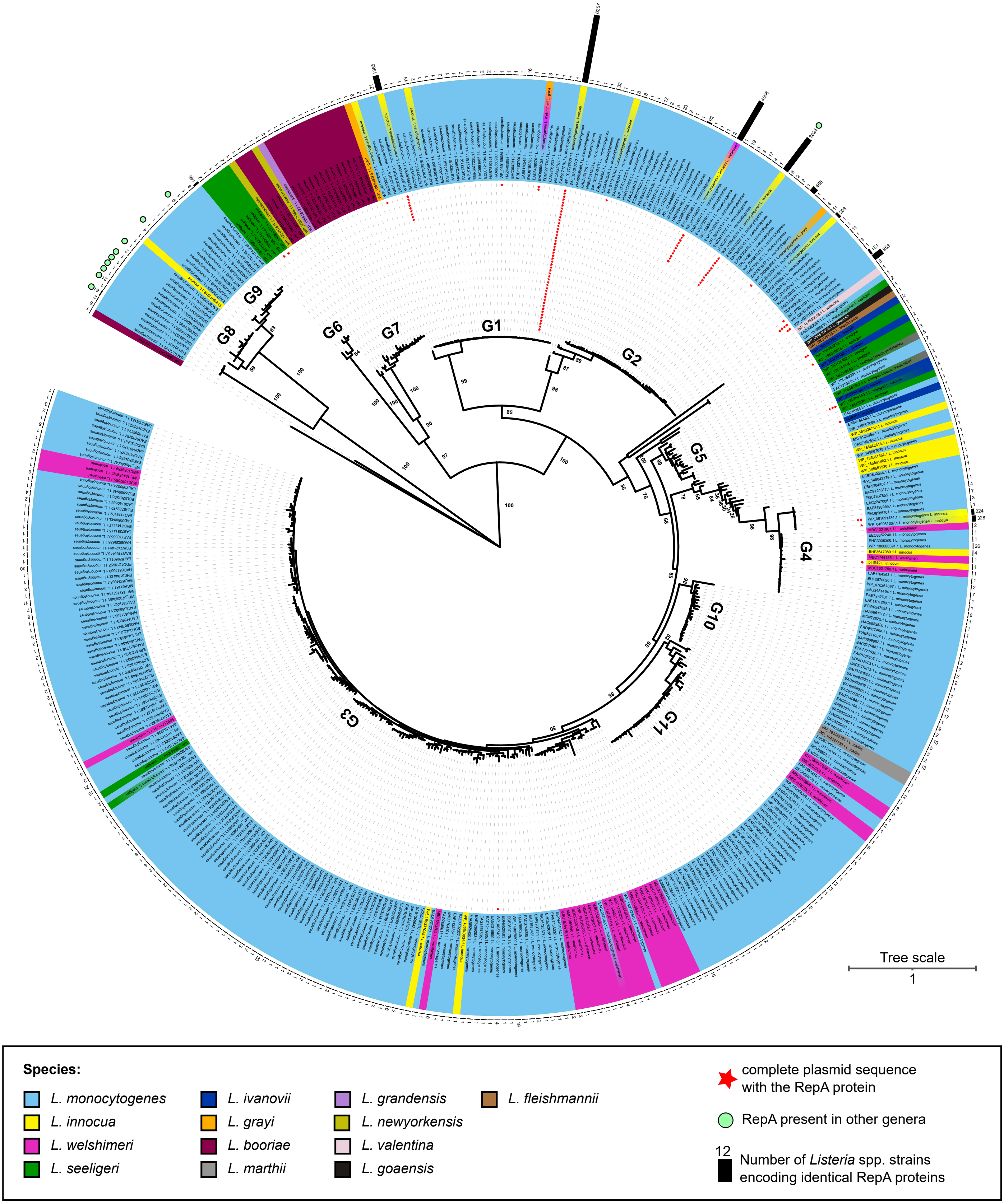

Figure S2. *Listeria* spp. RepA phylogenetic tree. Related RepA proteins were retrieved from the non-redundant Gen Bank database (24th April 2021) with a PSI-BLAST search of all *Listeria* spp. using WP_003728500.1 (RepA present in the highest number of complete plasmids). All sequences with "partial" in the description were removed. After manual inspection of alignments other putative partial sequences were also removed. Two unique RepA sequences were added from complete plasmid sequences obtained in this study. Next, identical sequences varying only by start-codon position annotation were identified, and after manual analyses at the nucleotide level duplicates were removed. A total of 418 unique RepA sequences were used for phylogenetic analyses. The tree was constructed in MEGAX based on 490 aa positions using the maximum-likelihood method (Le and Gascuel model). Statistical support for the internal nodes was determined by 100 bootstrap replicates. The tree was visualized in iTOL and drawn to scale, with branch lengths representing the number of substitutions per site. To estimate prevalence of the RepA proteins among *Listeria* species, records were retrieved from the Identical Protein Groups database for each RepA protein. Duplicated records (RefSeq/INSDC) with an identical assembly number withina RepA group were removed. The total number of strains harboring these RepA proteins (including strains harboring plasmids sequenced in this study) are marked on the outer ring. It should be noted that sequencing errors might result in additional, artifical variants of RepA proteins inflating the presented diversity. G1-G11 indicate main identified RepA groups.

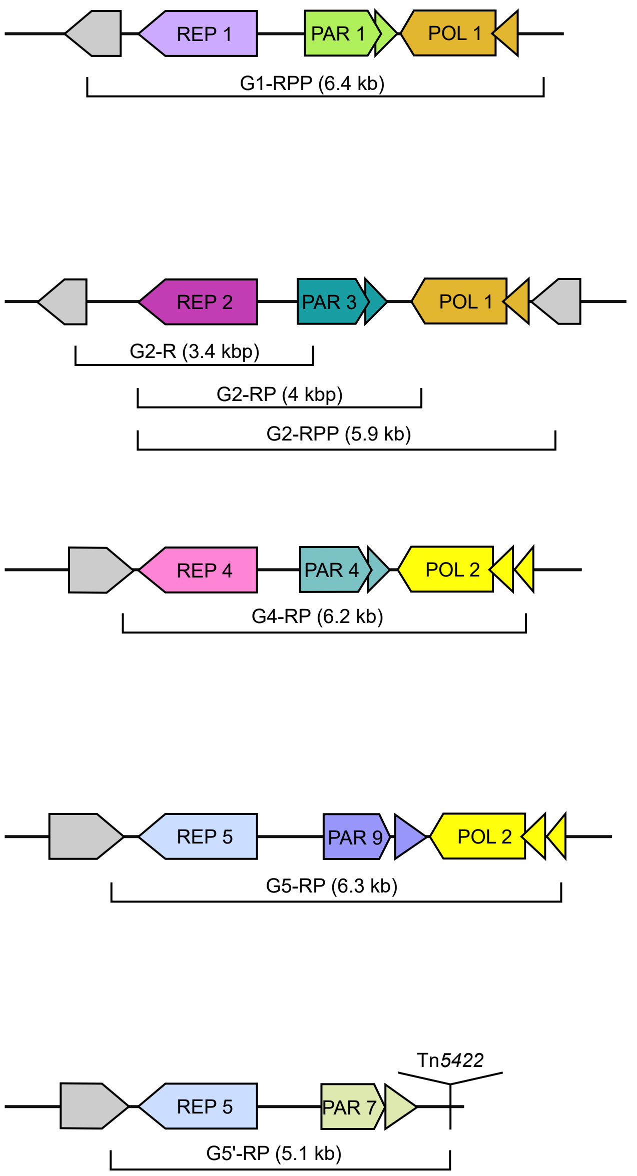

Plasmid	REP	PAR	POL	PRIMERS	RE	RE (pDKE2)
pLIS36				**F:** GGAGGTACC GCAACTTGTGAGCAAGAG<br>**R:** TAGCGGCCGC GCGCTAATAACCCAAGGACTAC	KpnI, NotI	KpnI, NotI
pLIS1				**F:** GCTCTAGA AGCGATGCAAGTTCTTCC<br>**R:** TAGAGCTC ACTTTGAGGGTCCAAGTC	XbaI, SacI	XbaI, SacI
				**F:** GCTCTAGA CAACAGCGAACAGTCAAAGG<br>**R:** TAGAGCTC GCGCTTATGCATGCGAATAG	XbaI, SacI	XbaI, SacI
				**F:** GCCATGCTAGC GGTAACAGCCCTAGTAAC<br>**R:** TAGCGGCCGC GCAACTTGTGAGCAAGAG	NheI, NotI	XbaI, NotI
pLIS26				**F:** TAGCGGCCGC CTATTCTCTGCGGTTCAC<br>**R:** GCGCTCTAGA ACCAGAAGTCGGTATTCC	NotI, XbaI	NotI, XbaI
pLIS6				**F:** GCTCTAGA TACAATGGGCAGCCGCAATAGG<br>**R:** TAGAGCTC CCACGTATGCAGGTGGTTATG	XbaI, SacI	XbaI, SacI
pLIS50				**F:** TAGCGGCCGC GTATCTCCACTGGGCAAATG<br>**R:** CTGCGCTCTAGA GCAATTATGGCGGATATGGG	NotI, XbaI	NotI, XbaI

Figure S3. Oligonucleotide primers and restriction enzymes used in cloning plasmid replication, partitioning and polymerase modules. RE - restriction enzyme

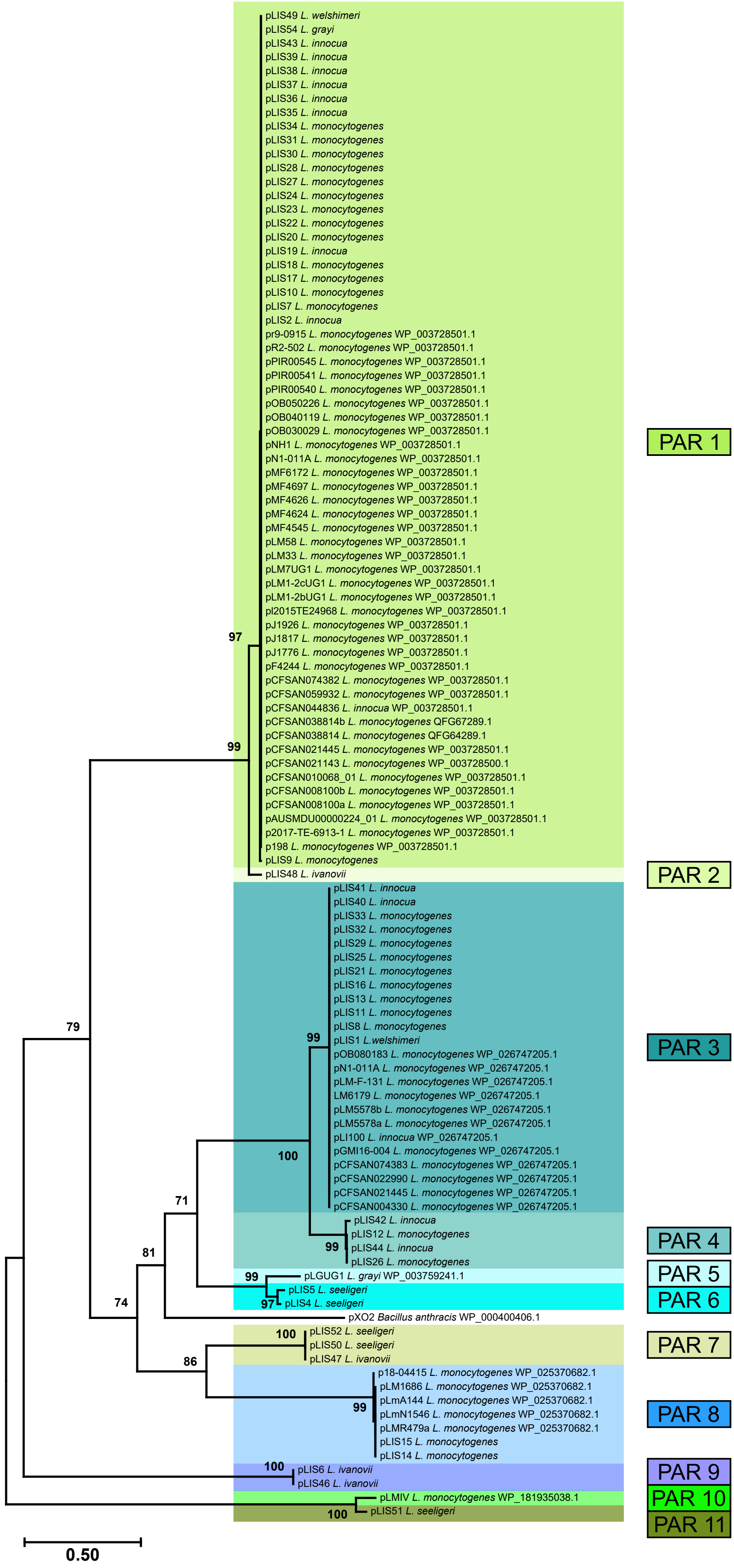

Figure S4. Phylogenetic tree of putative ParA proteins encoded by complete large *Listeria* spp. plasmid sequences and plasmid pXO2 of *Bacillus anthracis* (NZ_CP023003.1). The tree was constructed in MEGAX based on 257 amino acid positions (positions with <95% site coverage were eliminated), using the maximum-likelihood method (Le and Gascuel model). The tree is drawn to scale, with branch lengths representing the number of substitutions per site. Statistical support for the internal nodes was determined by 1000 bootstrap replicates.

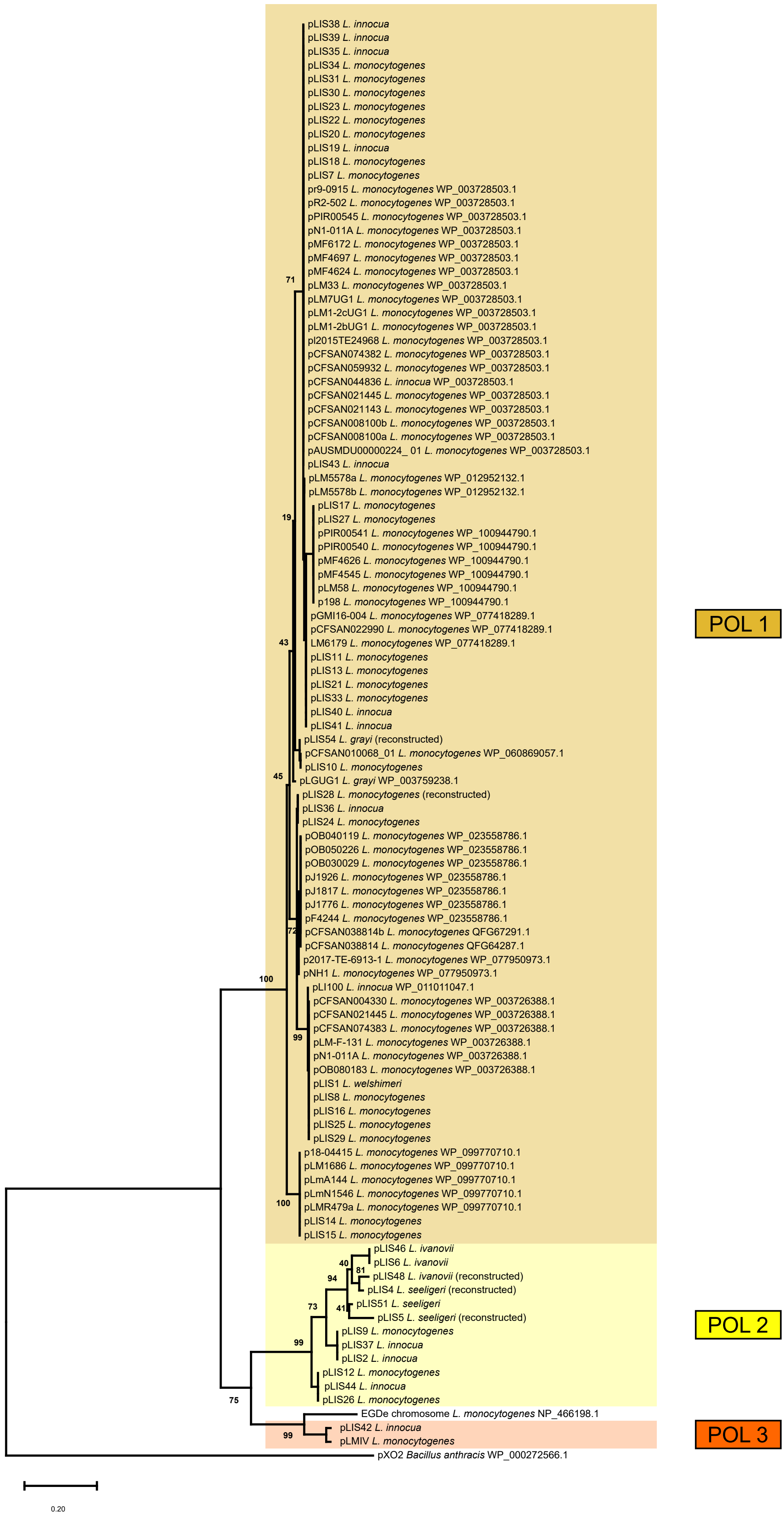

Figure S5. Phylogenetic tree of putative UvrX proteins encoded by complete large *Listeria* spp. plasmid sequences, *L. monocytogenes* EGDe chromosome (NC_003210.1) and plasmid pXO2 of *Bacillus anthracis* (NZ_CP023003.1) (as an out group). The tree was constructed in MEGAX based on 426 amino acid positions (positions with < 95% site coverage were eliminated), using the maximum-likelihood method (Le and Gascuel model). The tree is drawn to scale, with branch lengths representing the number of substitutions per site. Statistical support for the internal nodes was determined by 1000 bootstrap replicates. Reconstructed genes were used in the case of frameshifts or TE insertions.

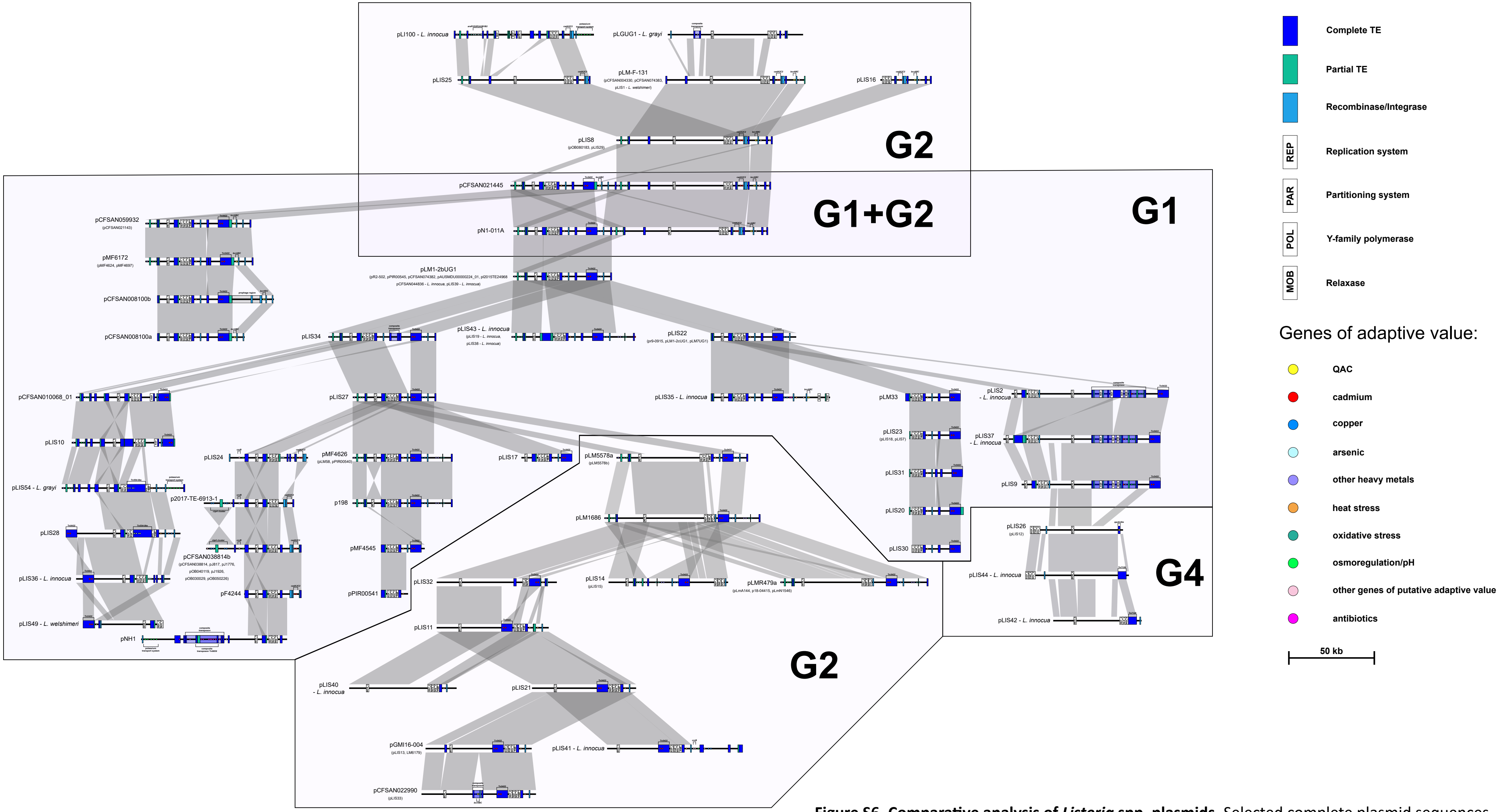

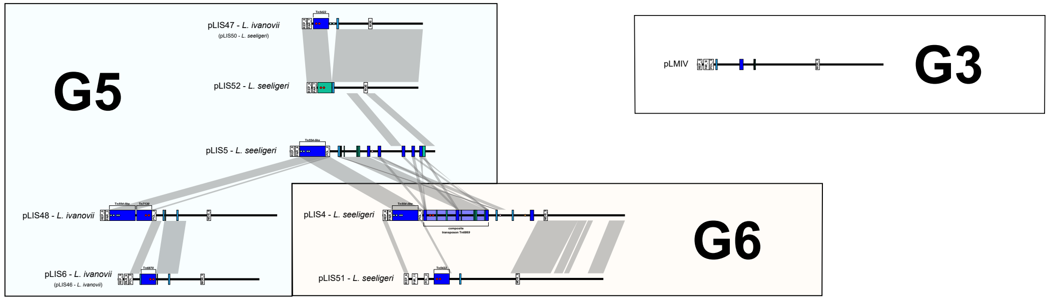

Figure S6. Comparative analysis of *Listeria* spp. plasmids. Selected complete plasmid sequences (available at GenBank or sequenced in our laboratory) were aligned and visualized using EasyFig. Regions of size >1 kb and nucleotide sequence identity > 90% are connected by gray boxes. Plasmids differing only by point mutations and small indels (of < 1 kb) are grouped together and represented by one plasmid of the largest size (names of others listed below). It should be noted that several plasmids located in distant parts of the alignment share highly similar regions, for which gray boxes were not shown in order for the visualization to remain readable. A simplified structure of the plasmids is presented, showing only the REP, PAR and POL modules, relaxases genes, genes of putative adaptive value as well as genetic elements that may play an important role in recombination events – i.e. TEs and recombinase genes. Unless indicated otherwise, plasmids originate from *L. monocytogenes* strains.

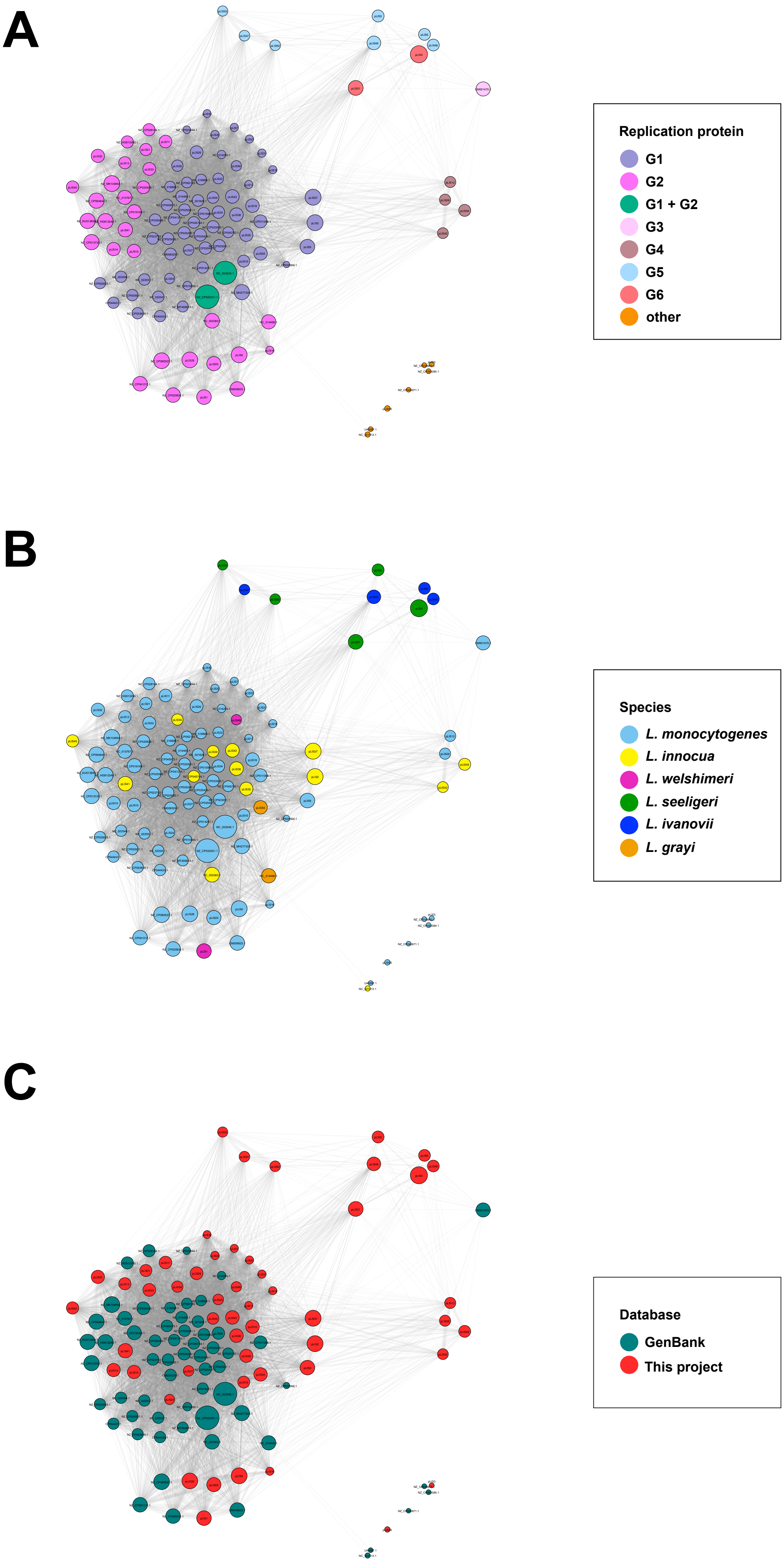

Figure S7. Protein-based similarity network of *Listeria* spp. plasmids. Each node (n=113) represents a complete plasmid sequence with the size of the node corresponding to the size of the plasmids. Edges reflect similarity between connected nodes. Colors correspond to (A) replication protein group, (B) host species, (C) sequence source.

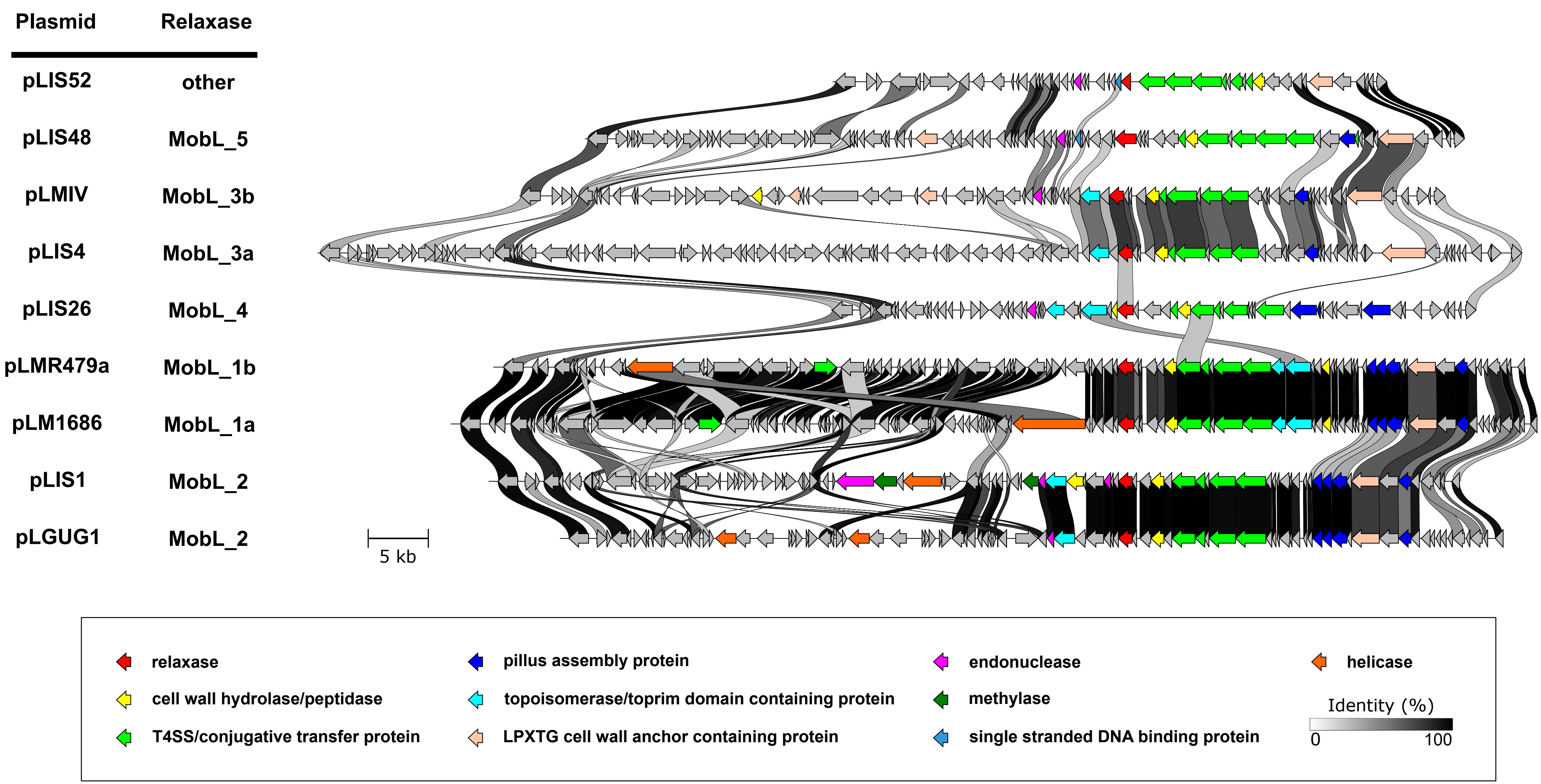

Figure S8. Comparison of putative conjugative transfer modules present in selected *Listeria* spp. plasmids; visualized with Clinker.
Gray shaded areas connect genes which encode proteins with >30% amino acid sequence similarity.

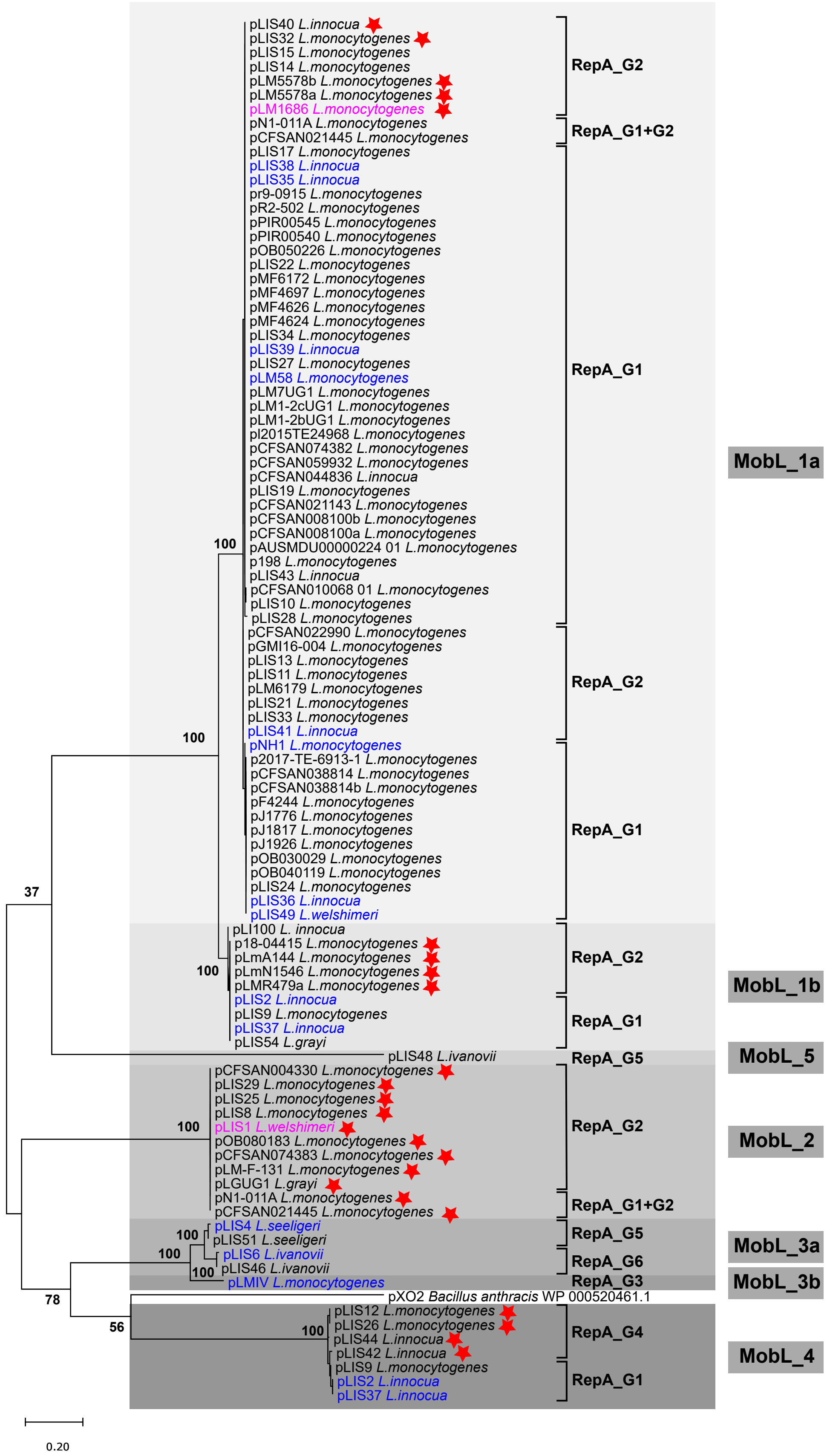

Figure S9. Phylogenetic tree of the MobL proteins encoded by complete large *Listeria* spp. plasmid sequences and plasmid pXO2 *Bacillus anthracis* (NZ_CP023003.1). The tree was constructed in MEGAX based on 371 amino acid positions, (positions with < 95% site coverage were eliminated), using the maximum-likelihood method (Le and Gascuel model). The tree is drawn to scale, with branch lengths representing the number of substitutions per site. Statistical support for the internal nodes was determined by 1000 bootstrap replicates. MobL relaxase genes adjacent to putative complete conjugative transfer modules (TRA) are marked with red stars. Plasmids which were successfully transferred in biparental mating experiments are highlighted in pink, while plasmids for which no transconjugants were obtained are highlighted in blue. The RepA groups of plasmids are indicated on the right.
